# Classifications within Molecular Subtypes Enables Identification of *BRCA1/BRCA2* Mutation Carriers by RNA Tumor Profiling

**DOI:** 10.1371/journal.pone.0064268

**Published:** 2013-05-21

**Authors:** Martin J. Larsen, Torben A. Kruse, Qihua Tan, Anne-Vibeke Lænkholm, Martin Bak, Anne E. Lykkesfeldt, Kristina P. Sørensen, Thomas v. O. Hansen, Bent Ejlertsen, Anne-Marie Gerdes, Mads Thomassen

**Affiliations:** 1 Department of Clinical Genetics, Odense University Hospital, Odense, Denmark; 2 Human Genetics, Clinical Institute, University of Southern Denmark, Odense, Denmark; 3 Epidemiology, Institute of Public Health, University of Southern Denmark, Odense, Denmark; 4 Slagelse Hospital, Department of Pathology, Slagelse, Denmark; 5 Department of Pathology, Odense University Hospital, Odense, Denmark; 6 Breast Cancer Group, Unit of Cell Death and Metabolism, Danish Cancer Society Research Center, Copenhagen, Denmark; 7 Center for Genomic Medicine, Rigshospitalet, University of Copenhagen, Copenhagen, Denmark; 8 Danish Breast Cancer Cooperative Group Statistical Center, Department of Oncology, Rigshospitalet, Copenhagen University Hospital, Copenhagen, Denmark; 9 Department of Clinical Genetics, Rigshospitalet, Copenhagen University Hospital, Copenhagen, Denmark; National Cancer Institute, National Institutes of Health, United States of America

## Abstract

Pathogenic germline mutations in *BRCA1* or *BRCA2* are detected in less than one third of families with a strong history of breast cancer. It is therefore expected that mutations still remain undetected by currently used screening methods. In addition, a growing number of *BRCA1*/2 sequence variants of unclear pathogen significance are found in the families, constituting an increasing clinical challenge. New methods are therefore needed to improve the detection rate and aid the interpretation of the clinically uncertain variants. In this study we analyzed a series of 33 *BRCA1*, 22 *BRCA2*, and 128 sporadic tumors by RNA profiling to investigate the classification potential of RNA profiles to predict *BRCA1/2* mutation status. We found that breast tumors from *BRCA1* and *BRCA2* mutation carriers display characteristic RNA expression patterns, allowing them to be distinguished from sporadic tumors. The majority of *BRCA1* tumors were basal-like while *BRCA2* tumors were mainly luminal B. Using RNA profiles, we were able to distinguish *BRCA1* tumors from sporadic tumors among basal-like tumors with 83% accuracy and *BRCA2* from sporadic tumors among luminal B tumors with 89% accuracy. Furthermore, subtype-specific *BRCA1/2* gene signatures were successfully validated in two independent data sets with high accuracies. Although additional validation studies are required, indication of *BRCA1/2* involvement (“BRCAness”) by RNA profiling could potentially be valuable as a tool for distinguishing pathogenic mutations from benign variants, for identification of undetected mutation carriers, and for selecting patients sensitive to new therapeutics such as PARP inhibitors.

## Introduction

Breast cancer is the most frequent malignant disease and the leading cause of cancer death among women [1]. It is estimated that approximately 5 to 10% of all breast cancers have a strong hereditary component. The families of these patients often show an apparently dominant inheritance pattern of breast cancer and are often characterized by early age of onset and presence of ovarian cancer, bilateral breast cancer, and male breast cancer. Germline mutations in *BRCA1* and *BRCA2* are detected in up to 28% of these breast-cancer families; however, it is expected that mutations still remain undetected by the currently used screening methods [2], [3]. In addition, a recent study has demonstrated that as many as half of mutation carriers lack an obvious family history and will therefore not be identified by current selection criteria [4]. Identification of a pathogenic mutation enables pre-symptomatic mutation testing of healthy family members. Female carriers of *BRCA1* or *BRCA2* mutations have a lifetime risk of 49 to 87% for developing breast cancer [5], [6], wherefore they are offered intensive cancer surveillance as well as risk reducing surgery. Sequence variants with uncertain pathogenicity (e.g., missense mutations, in-frame indels, and splice site mutations) are frequently found in *BRCA1* and *BRCA2*, but the clinical significance of these variants is often unknown and therefore implies an additional clinical challenge. With the forthcoming implementation of next-generation sequencing methods in many diagnostic settings, the number of clinical uncertain variants will increase significantly and result in a major clinical challenge. Therefore, new methods are needed to aid in the interpretation of uncertain variants as well as to increase the detection rate of *BRCA1* and *BRCA2* germline mutations for genetic counseling and clinical management of familial breast cancers.

The histopathological characteristics of *BRCA1* and *BRCA2* tumors are well described. *BRCA1* tumors are frequently high-grade (grade 3), ductal carcinomas with necrotic areas and lymphocytic infiltration. In addition, higher frequency of medullary carcinomas has been observed among *BRCA1* tumors compared to sporadic tumors. Most *BRCA2* tumors are grade 2/3, ductal carcinomas with high mitotic rates [7], [8]. *BRCA1* tumors are typically estrogen receptor (ER) negative, progesterone receptor (PR) negative and HER2 negative (triple-negative) cancers, while the majority of *BRCA2* tumors are ER positive and HER2 negative [9]. None of these features is, however, unique and therefore none can be used to distinguish *BRCA1* and *BRCA2* tumors from sporadic breast tumors.

Microarray-based gene expression profiling of breast cancers have revealed the existence of at least four clinically relevant subgroups, designated basal-like, HER2-enriched, luminal A (lumA), and luminal B (lumB) [10]–[13]. The molecular subtypes correspond broadly to histopathological characteristics and are associated with different clinical outcomes. Basal-like cancers are mostly high-grade, triple-negative tumors with high expression of basal epithelial markers such as CK5/14/17; while HER2-enriched cancers are associated with amplification of the HER2-amplicon. LumA cancers are typically low-grade, ER+ tumors while lumB are high-grade, ER+ cancers. Cancers of the luminal subtypes show high expression of luminal-associated genes such as CK8/18. In addition to these four subtypes, a normal-like subtype has also been identified which shows high similarity to normal breast epithelium. Whether the normal-like tumor type represents an independent tumor subtype or just reflects low amounts of tumor cells in the biopsy is currently not clear. Array-CGH and next-generation sequencing studies have demonstrated that the molecular subtypes are associated with distinct patterns of copy number aberrations and somatic genomic rearrangements [14]–[17].

Although numerous RNA profiling studies of breast cancers have been published, only a limited number of studies of breast tumors from *BRCA1* and *BRCA2* mutation carriers exist [14], [18]–[22]. In general, these studies are small in terms of sample size due to limited access to frozen tumor tissue and/or conducted on early-generation microarray platforms. The more recent studies have specified that tumors from *BRCA1* mutation carriers are primarily basal-like while the majority of *BRCA2* tumors are of luminal subtypes [14], [21]–[23]. Due to limitations in study designs, only a few studies have investigated the classification potential in relation to *BRCA1/2* mutation status [18]–[20]. Although some studies reported fairly high BRCA1 classification accuracies, there has been some concern, as the results may have been confounded due to lack of proper sample matching [24]. To our knowledge, none of the published *BRCA1/2* signatures have ever been validated. Array-CGH analyses have indicated that *BRCA1/2* tumors show characteristic genomic patterns, which have been used for classification with varying results [21], [25]–[27].

Early-phase clinical studies have indicated promising effects of poly(ADP-ribose) polymerase (PARP) inhibitors among *BRCA1/2* mutation carriers due to dysfunctional DNA repair by homologous recombination (HR) [28], [29]. Other molecular mechanisms, such as mutations in *BRCA*-related genes or promoter hyper-methylation, might also lead to *BRCA*-associated HR-deficiencies, and such tumors might be sensitive to PARP inhibitors. However, a recent phase-III trial among a cohort of triple-negative-breast cancer patients with unknown *BRCA1/2* status failed to show prolonged survival [30]. This calls for a better definition of the tumor phenotype for better prediction of response and patient selection. Therefore, development of new methods for improved identification and clarification of *BRCA*/HR-deficiency will not only provide more accurate risk-assessments in genetic counseling but may also be used to determine optimal treatment strategies.

In the present study, we have performed microarray gene expression profiling for molecular characterization and classification of *BRCA1*, *BRCA2*, and sporadic (unselected) breast cancers. Gene expression-based identification of *BRCA*-associated breast cancers could have various clinical applications including identification of mutation carriers that are undetected by currently used methods, evaluation of the sequence variants of unknown clinical significance, and selection of patients sensitive to new therapy regimens.

## Materials and Methods

### Ethics statement

The study has been approved by the Danish Ethical Committee System (S-VF-20020142), waiving the requirement for informed consent for the study.

### Patient material

The study was performed on frozen primary breast-tumor samples collected between 1982 and 2008. The samples were obtained from the bio-banks of the Dept. of Pathology, Odense University Hospital and the Danish Breast Cancer Cooperative Group (DBCG). Breast tumors from hereditary breast-cancer patients carrying a known pathogenic *BRCA1* (*n = *33) or *BRCA2* (*n = *22) germline mutation were included in the study. Serving as a representative control group, primary breast-tumor samples (*n = *128) were randomly selected among available samples originating from the same department and time period as for the hereditary samples. The family histories of the control patients were unknown, but none of the patients had been referred to genetic counseling at Odense University Hospital, where the vast majority of patients were recruited, and are therefore here referred to as sporadic. In total, 183 tumor samples were analyzed. Tumor and patient characteristics are summarized in Table 1.

**Table 1:** Patient and tumor characteristics.

			BRCA1 (*n = *33)	BRCA2 (*n = *22)	Sporadic (*n* = 128)
**Estrogen receptor**					
		ER+	14	20	107
		ER−	19	2	21
**Progesterone receptor**					
		PR+	7	16	79
		PR−	26	6	49
**HER2 status**					
		HER2+	3	1	21
		HER2−	30	21	107
**Lymph node**					
		LN+	15	14	51
		LN−	16	7	75
		NA	2	1	2
**Tumor size**					
		Mean tumor size, mm (±SD)	23 (±10)	25 (±13)	25 (±16)
**Histologic grade**					
		Grade 1	3	2	32
		Grade 2	7	11	48
		Grade 3	18	7	29
		NA	5	2	19
**Tumor type**					
		Invasive ductal carcinoma	28	19	105
		Invasive lobular carcinoma	1	2	12
		Mucinous carcinoma	0	0	2
		Medullary carcinoma	2	0	1
		Tubular carcinoma	0	0	3
		Metaplastic carcinoma	0	0	0
		Other	0	0	2
		NA	2	1	3
**Age**					
		Median age, years (range)	42 (25–74)	43.5 (28–72)	61 (27–95)
		<50 years	21	15	21
		≥50 years	12	7	107
**Menupause status**					
		Premenopausal	20	15	30
	Perimenopausal		0	1	15
	Postmenopausal		12	5	78
	Other		0	0	2
	NA		1	1	3

### Histopathological review

Samples included in the study contained at least 50% tumor cells determined by representative haematoxylin-eosin-stainings. Histopathological data and ER and PR, and HER2 statuses determined by immunohistochemical (IHC) were obtained from DBCG. Furthermore, gene-expression levels of *ESR1*, *PGR*, and *ERBB2* were used to determine ER, PR, and HER2 status, respectively. Cut-off levels were optimized using available IHC data (Figure S1).

### Gene-expression analysis

Total RNA was extracted from freshly frozen tumor tissue using Trizol Reagent (Invitrogen) and RNeasy Micro Kit (Qiagen). RNA concentration was determined using a NanoDrop, and the quality was assessed by the Agilent 2100 Bioanalyzer. RNA samples used in the study had RIN scores ranging from 5.9 to 9.6. Gene-expression analysis was performed using a customized version of Agilent SurePrint G3 Human GE 8×60K Microarray (Agilent Technologies). RNA was amplified and labeled using the Amino Allyl MessageAmp II aRNA Amplification Kit (Ambion) according to the manufacturer's protocol. Amplified aRNA from the tumor samples were labeled with Cy5. Universal Human Reference RNA (Stratagene) was labeled with Cy3 and used as a reference. Hybridization, washing, scanning, and quantification were performed according to the array manufacturer's recommendations.

### Data pre-processing

Raw intensity data were background corrected using normexp method, within-array normalized by loess method and between-array normalized by the quantile method [31], [32]. Finally, log_2_-transformed Cy5/Cy3 ratios were obtained, replicate probes were collapsed by calculating the median, and probes without gene-symbol annotation were filtered out. In cases of multiple probes per gene symbol, only the probe with the highest Cy5 mean intensity was kept. Data pre-processing was performed using the R package *limma*. Microarray data have been deposited to the Gene Expression Omnibus (GSE40115).

### Unsupervised methods

Unsupervised hierarchical clustering (Euclidian metric, complete linkage) and principal-component analysis (PCA) were carried out in Qlucore Omics Explorer. Expression levels of each gene had been standardized to zero mean and unit variance.

### Molecular subtype classification

The 50-gene subtype classifier described by Parker et al. was used to classify tumors into five intrinsic molecular subtypes [12]. Distances to each of the subtype centroids defined by the PAM50 classifier were calculated using Spearman's rank correlation using the R package *genefu*; hereby the subtype classification was assigned based on the nearest of the centroids.

### Classification of *BRCA1* and *BRCA2* breast cancers

For classification of *BRCA1*, *BRCA2*, and sporadic breast tumors, the support vector machines (SVM) implementation found in the R package *e1071* was applied with linear kernel. The classifications were performed using the leave-one-out cross-validation (LOOCV) method, as it provides an unbiased performance estimate. In each iteration, one sample was held out and the remaining samples were used for training. The trained model was then tested on the left-out sample and the result was compared to the true class in order to estimate accuracy. The procedure was repeated until each of the samples had been left out once. During each LOOCV round, an optimized gene set was selected by first ranking the genes according to their *t*-statistics (Welch's *t*-test), using only the training samples, and the optimal number of top-genes was found by step-wise increasing the number of genes from the top of the ranked list; at each increment the classification accuracy of the training samples was assessed using LOOCV in a nested loop. To account for unequal group sizes, the SVM probability estimate was adjusted according to the group sizes. Mean balanced accuracy was used as a performance measure (mean of sensitivity and specificity). The significance of the classification results was calculated using Fisher's exact test on 2×2 contingency tables.

### Development of gene signatures

Due to the nature of the gene-selection procedure described above, different gene sets were selected in each of the LOOCV iterations, resulting in the same number of gene sets as the number of samples. In order to identify specific gene signatures, genes were ranked according to their *t*-statistics using all samples. The top-ranked differentially expressed genes were used to define the gene signatures. The number of predictive genes to be included was optimized by the LOOCV procedure.

### Validation of gene signatures

Cross-platform validation of the gene signatures was conducted using a subset of the tumor samples analyzed by our in-house spotted microarray platform [33]. External validation was performed using data sets from Netherlands Cancer Institute (NKI) by van't Veer et al. and Lund University by Jönsson et al. [14], [19]. Preparation procedures of the in-house spotted data set and the two independent data sets are described in the supplementary information (see Methods S1). Performances of the gene signatures in the validation data sets were estimated by LOOCV using SVM.

## Results

### Pathological characteristics of patient material

In the present study, frozen, primary breast tumors were collected from *BRCA1* (*n = *33) and *BRCA2* (*n = *22) mutation carriers and from sporadic cases (*n = *128). Tumor and patient characteristics are summarized in Table 1. Median age of diagnosis was 42 years among *BRCA1*, 43.5 years among *BRCA2* mutation carriers, and 61 years for sporadic breast cancer patients. The patient material consisted mainly of ductal carcinomas, with a minor fraction of lobular carcinomas. Among sporadic tumors, tumor grades were found more evenly distributed. Eighteen (55%) of the 33 *BRCA1* tumors displayed the triple-negative phenotype (ER−/PR−/HER2−), compared with only 10 of the 128 (8%) sporadic tumors. Tumors obtained from *BRCA2* carriers were predominantly ER+ (91%), PR+ (73%), and HER2− (95%).

### Unsupervised hierarchical clustering

**Figure 1:**
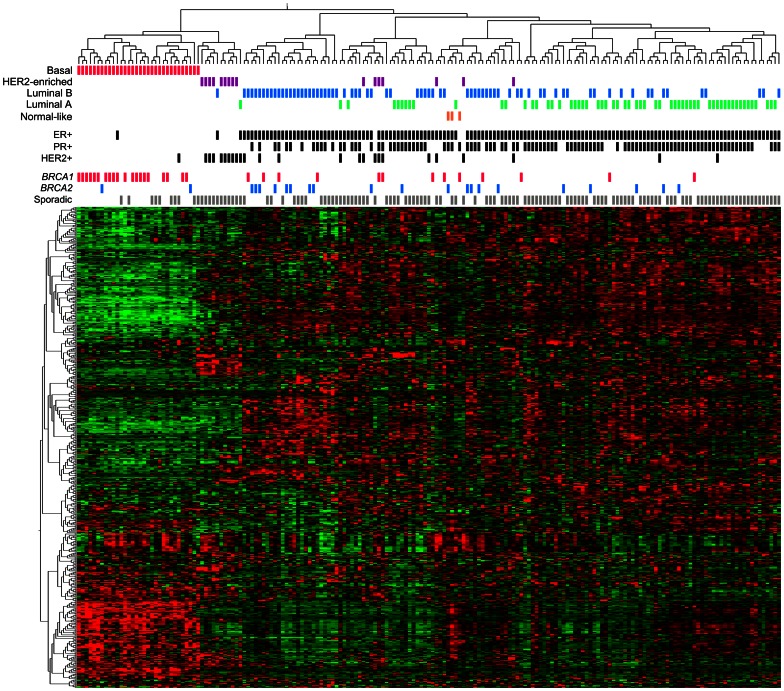
Hierarchical clustering. Hierarchical clustering of 183 breast tumor samples using the 500 most variant genes across all samples. In the heat map rows correspond to genes and columns to samples. Red indicates elevated expression, green reduced expression.

Unsupervised hierarchical clustering of the 183 tumor samples using the 500 most variant genes out of 22,171 probes with unique gene symbols assigned resulted in the formation of two main branches clearly separating ER− from ER+ tumors (Figure 1). The ER− cluster could be further divided into two sub-clusters; a triple-negative cluster, containing a large fraction of the *BRCA1* tumors; and a HER2+/ER−/PR− cluster. Tumors were classified into intrinsic molecular subtypes (basal-like, lumA, lumB, HER2-enriched, or normal-like) by the PAM50 classifier proposed by Parker et al. [12]. The triple-negative cluster represents exclusively basal-like tumors, while the HER2+/ER−/PR− cluster contained primarily tumors of the HER2-enriched subtype. The vast majority of luminal tumors were found within the ER+ cluster, including most *BRCA2* and ER+ *BRCA1* tumors.

### Molecular subtypes of hereditary breast cancer

**Figure 2:**
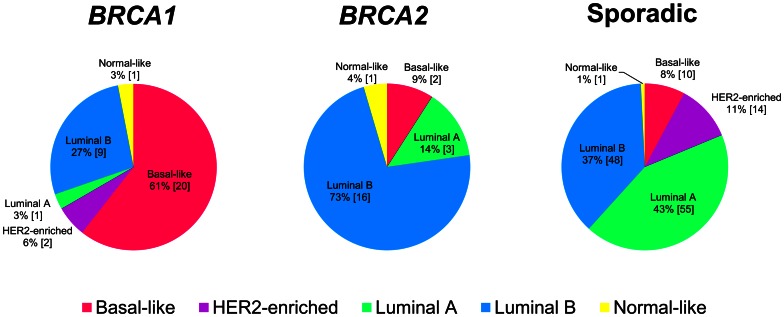
Association between hereditary breast cancers and molecular subtypes. Distribution of molecular subtypes among *BRCA1*, *BRCA2* and sporadic breast cancer samples. Tumors were classified into molecular subtypes using the PAM50 classifier. Numbers in brackets refer to number of samples in each group.

The distribution of the predicted intrinsic molecular subtypes within *BRCA1*, *BRCA2*, and sporadic tumors was determined (Figure 2, Tables S1, S2, S3, and S4). *BRCA1* tumors were associated with the basal-like subtype (p = 4×10^−10^, Fisher's exact test), while *BRCA2* tumors were associated with the lumB subtype (p = 4×10^−3^, Fisher's exact test). Among sporadic tumors the subtypes were more evenly distributed but with the majority being lumA (43%) and lumB (37%). The HER2-enriched subtype was absent in *BRCA2* tumors, and only two *BRCA1* tumors were HER2-enriched.

### General *BRCA1* and *BRCA2* classification

The aim of the project was to explore whether RNA profiles can be used to identify tumors from *BRCA1* and *BRCA2* mutations carriers. Our initial approach was to conduct general classifications, not taking other clinically relevant subgrouping into consideration. Classifications were performed using LOOCV. Conducting *BRCA1*-*versus*-sporadic classification, 23 out of 33 *BRCA1* tumors were correctly classified as *BRCA1*, while 109 of the 128 sporadic tumors were classified correctly (Table 2). Consequently, the balanced accuracy was 77% (sensitivity: 70%, specificity: 85%). *BRCA2*-*versus*-sporadic classification correctly predicted 16 out of 22 *BRCA2* tumors and 109 out of 128 sporadic tumors, resulting in a balanced accuracy of 83% (sensitivity: 82%; specificity: 85%).

**Table 2:** General classification and within-subtype classification of BRCA1 and BRCA2 breast cancers.

	No. of samples	Sensitivity (TP)	Specificity (TN)	Accuracy^a^	*p*-value^b^
**General classification**					
All: *BRCA1* vs. sporadic	33 vs. 128	0.70 (23)	0.85 (109)	0.77	2.3×10^−9^
All: *BRCA2* vs. sporadic	22 vs. 128	0.82 (18)	0.85 (109)	0.83	9.0×10^−10^
**Within-subtype classification**					
Basal: *BRCA1* vs. sporadic	20 vs. 10	0.85 (17)	0.80 (8)	0.83	1.0×10^−3^
LumB: *BRCA1* vs. sporadic	9 vs. 48	0.44 (4)	0.79 (38)	0.62	2.0×10^−1^
LumB: *BRCA2* vs. sporadic	16 vs. 48	0.88 (14)	0.90 (43)	0.89	2.4×10^−8^

Classification performances were assessed by leave-one-out cross-validation. TP, true positive; TN, true negative.

a Mean balanced accuracy.

b Fisher's exact test.

As *BRCA1* and *BRCA2* tumors are known to be unequally distributed between the molecular subtypes, we evaluated the classification performances of the general classifications within each of the molecular subtypes to investigate whether the classifications were influenced by subtypes (Table S5). Among basal-like tumors, 19 out of 20 basal-like *BRCA1* tumors were correctly classified;; but unfortunately all sporadic tumors were misclassified as *BRCA1* (balanced accuracy: 48%, sensitivity: 95%, specificity: 0%). Within the group of lumB tumors, only 3 out of 9 *BRCA1* tumors were classified correctly, while 44 out of 48 sporadic tumors were classified correctly (balanced accuracy: 63%, sensitivity: 33%, specificity: 92%). Investigating the *BRCA2* classification in the context of molecular subtypes revealed that 14 out of 16 lumB *BRCA2* tumors were classified correctly, whereas 9 out of 48 sporadic tumors were misclassified as *BRCA2* (balanced accuracy: 84%, sensitivity: 88%, specificity: 81%). Four out of 6 non-lumB *BRCA2* samples and 70 out of 80 non-lumB sporadic samples were classified correctly (balanced accuracy: 77%, sensitivity: 67%, specificity: 88%).

### Within-subtype *BRCA1* and *BRCA2* classification

**Figure 3:**
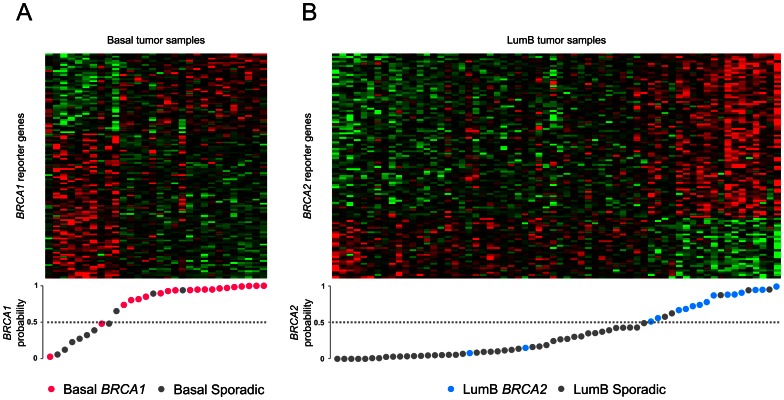
Within-subtype classification of basal *BRCA1* and lumB *BRCA2* breast cancers. Expression data matrix of the 110-gene basal *BRCA1* signature (A) and the 100-gene lumB *BRCA2* signature (B) are visualized as heat maps. Rows correspond to genes and columns to samples. Tumors are ordered according to their *BRCA1/2* probability estimate obtained by leave-one-out cross-validation (lower panels). The germline mutation is shown as red (*BRCA1*), blue (*BRCA2*) or grey (sporadic). Dashed lines indicate the *BRCA1/2* probability cutoff. Samples with probabilities ≥0.5 are classified as *BRCA1/2*, while samples with probabilities <0.5 are classified as sporadic tumors.

The general classification approach appeared to be influenced by molecular tumor subtypes. This was especially true for the *BRCA1* classification, for which all sporadic basal-like tumors were misclassified as *BRCA1*. To avoid any potential confounding effects of the tumor subtypes, we wanted to test whether stratification by molecular subtypes could improve *BRCA1/2* classifications. The majority of *BRCA1* tumors were found to be either basal-like or lumB. Thus, *BRCA1* classifications were conducted within basal-like samples and within lumB samples, respectively. Subtype stratification improved the *BRCA1* classification markedly among basal-like tumors. Using this procedure, now 17 out of 20 basal-like *BRCA1* tumors and 8 out of 10 basal-like sporadic tumors were correctly classified (balanced accuracy: 83%, specificity: 85%, sensitivity: 80%) (Figure 3 and Table 2). Compared with the performance of the general *BRCA1* classification this represents a 35% increase in balanced accuracy among the basal-like tumors. LumB *BRCA1* classification resulted in a balanced accuracy of 62% (sensitivity: 44%, specificity: 79%), which is comparable to the performance of the general *BRCA1* classification among lumB tumors. Likewise, *BRCA2* classification was performed among lumB tumors, as the vast majority of *BRCA2* tumors were of the lumB subtype. This resulted in a balanced accuracy of 89% (sensitivity: 88%, specificity: 90%), which represents a 5% increase relative to the general *BRCA2* classification within lumB tumors. Details of the classification results can be found in Tables S6, S7, and S8.

### Subtype-specific gene signatures

The LOOCV classification scheme resulted in slightly different gene sets in each round of cross-validation. In order to obtain specific gene signatures for validation purposes, genes were ranked according to their *t*-statistics, and the top-ranked differentially expressed genes were used to define the signatures. LOOCV was used to determine the optimal lengths of the gene lists/signatures. This resulted in identification of a 110-gene basal *BRCA1* gene signature and a 100-gene lumB *BRCA2* signature (Figure 3, Figure S2, S3, and Table S9, S10).

### Cross-platform reproducibility of gene-expression signatures

To evaluate the reproducibility of the expression patterns of the signatures, a subset of the tumor samples was analyzed using our in-house spotted microarray platform. From the basal *BRCA1* signature 95/110 genes were present on the spotted platform, while 92/100 genes from the lumB *BRCA2* signature could be identified. Using LOOCV, we obtained balanced accuracies of 93% for the *BRCA1* signature and 96% for the *BRCA2* signature (Table 3).

**Table 3:** Cross-platform validation of the gene signatures.

Gene signature	No. of samples	Overlapping genes	Sensitivity (TP)	Specificity (TN)	Accuracy^a^	*p*-value^b^
**Basal ** ***BRCA1*** ** signature**	20 vs. 10	95/110	0.95 (19)	0.90 (9)	0.93	6.7×10^−6^
**LumB ** ***BRCA2*** ** signature**	16 vs. 46	92/100	0.94 (15)	0.96 (44)	0.96	2.2×10^−11^

Validation of the basal *BRCA1* signature and lumB *BRCA2* signature were performed using samples analyzed by in-house spotted microarrays. Classification performances were assessed by leave-one-out cross-validation. TP, true positive; TN, true negative.

a Mean balanced accuracy.

b Fisher's exact test.

### Validation in independent data sets

**Figure 4:**
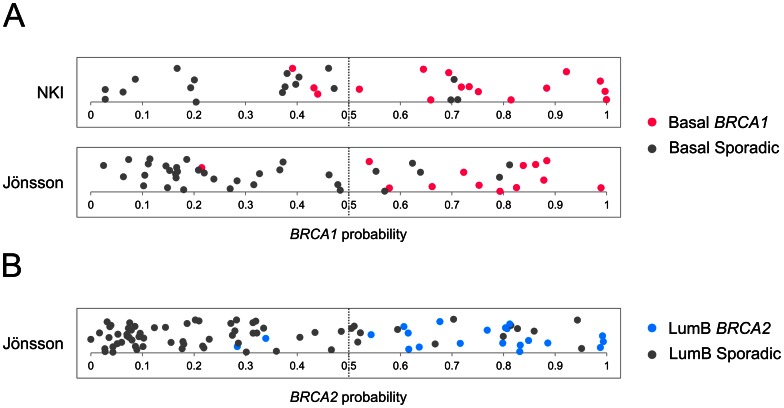
Validation of the basal *BRCA1* signature and lumB *BRCA2* signature in independent datasets. A) The basal *BRCA1* signature was validated using basal-like tumor samples obtained from the NKI dataset and Jönsson dataset, respectively. The panels show the *BRCA1* probability estimates of basal-like *BRCA1* samples (red) and basal-like sporadic samples (grey). B) The lumB *BRCA2* signature was validated using lumB tumor samples obtained from the Jönsson dataset. The panel shows the *BRCA2* probability estimates of lumB *BRCA2* samples (blue) and lumB sporadic samples (grey). Probability estimates were obtained by leave-one-out cross-validation. Dashed lines indicate the *BRCA1/2* probability cutoff. Samples with probabilities ≥0.5 are classified as *BRCA1/2*, while samples with probabilities <0.5 are classified as sporadic tumors. Samples have been “jittered” in the vertical direction to spread them out for better visualization.

To test the general classification validity of the gene signatures, the signatures were also tested in two independent data sets, the NKI data set published by van't Veer et al. [19] and the Jönsson et al. data set [14]. To obtain the most valid and up-to-date gene-symbol annotation, probe-information was re-annotated. Initially, samples were classified according to their molecular subtypes by applying the PAM50 classifier. This revealed 16 *BRCA1* and 18 sporadic basal-like tumors in the NKI data set. In the Jönsson data set, 13 *BRCA1* and 34 sporadic basal-like tumors were found, as well as 21 *BRCA2* and 68 sporadic lumB tumors (Table S11). The NKI data set contained only two *BRCA2* tumors, both classified as lumA (Table S12). Seventy-six of the 110 genes from the basal *BRCA1* signature were contained on the Rosetta chip used in the NKI study, while 69 genes were present in the Jönsson data set. The performance of the signature was estimated by LOOCV, using the SVM algorithm. *BRCA1* classification among the basal-like samples in the NKI data set resulted in a balanced accuracy of 82% (sensitivity: 81%, specificity: 83%) (Figure 4 and Table 4). Likewise, *BRCA1* status was predicted in the Jönsson data set with 87% balanced accuracy (sensitivity: 93%, specificity: 82%). The lumB *BRCA2* signature was tested in the Jönsson data set, where 77 out of the 100 genes were present, resulting in a balanced accuracy of 87% (sensitivity: 90%, specificity: 83%).

**Table 4:** Validation of gene signatures in independent datasets.

	No. of samples	Overlapping genes	Sensitivity (TP)	Specificity (TN)	Accuracy^a^	*p*-value^b^
**Basal ** ***BRCA1*** ** signature**						
NKI dataset	16 vs. 18	76/110	0.81 (13)	0.83 (15)	0.82	3.9×10^−4^
Jönsson dataset	13 vs. 34	69/110	0.93 (12)	0.82 (28)	0.87	3.9×10^−6^
**LumB ** ***BRCA2*** ** signature**						
Jönsson dataset	21 vs. 68	77/100	0.90 (19)	0.83 (57)	0.87	7.3×10^−10^

The basal BRCA1 signature and lumB BRCA2 signature was validated in two public available datasets. Classification performances were assessed by leave-one-out cross-validation. TP, true positive; TN, true negative.

a Mean balanced accuracy.

b Fisher's exact test.

## Discussion

In the current study, we have characterized breast tumors from female carriers of germline mutations in *BRCA1* and *BRCA2* genes and a cohort of sporadic (unselected) breast tumors by microarray gene-expression analysis. We have developed molecular signatures that can be used to distinguish *BRCA1* and *BRCA2* tumors from sporadic tumors with high accuracy. This approach has potential as a functional assay in the current genetic diagnostic to indicate BRCA1/2 involvement, which could be useful in the interpretation of sequence variants with unknown clinical significance and for treatment stratification. Furthermore, we have shown that specific histopathological characteristics and molecular subtypes are associated with *BRCA1/2* tumors.

### *BRCA1/2* mutations and their relation to specific histopathological characteristics and molecular subtypes

We have shown that *BRCA1* tumors were more frequently ER− compared with sporadic tumors. In contrast, the vast majority of tumors arising in *BRCA2* carriers were ER+, and only very few of the *BRCA1/2* positive breast tumors demonstrated HER2-amplification. These histopathological characteristics of *BRCA1* and *BRCA2* tumors included in our study are in accordance with a recent study by the CIMBA consortium in which the pathology of 4,325 *BRCA1* and 2,568 *BRCA2* mutation carriers have been described [9].

The histopathological characteristics of the tumors were clearly reflected in their molecular subtypes, as *BRCA1* tumors were primarily basal-like or lumB while *BRCA2* tumors were predominantly classified as lumB. We found the group of basal-like tumors to be highly overlapping with the group of triple-negative tumors. Out of 30 triple-negative tumors, 29 were classified as basal-like (97%). Conversely, 29 of the 33 basal-like tumors were triple-negative (88%). The distribution of molecular subtypes among tumors from *BRCA1* and *BRCA2* mutations carriers has only been assessed in a few other studies and with frequencies comparable to our observations [14], [21], [22].

The pronounced association between *BRCA1/2* mutations and specific molecular subtypes strongly indicates that mutation carriers are, not only predisposed to develop breast cancer, but also to develop specific subtypes of breast cancer. Both *BRCA1* and *BRCA2* are implicated in mediating repair of double-strand breaks by homologous recombination (HR). Cells with impaired function of *BRCA1* and *BRCA2* are unable to repair double-strand breaks by the error-free HR, resulting in repair by the error-prone non-homologous end-joining (NHEJ) pathway [34], [35]. The function of *BRCA2* is probably restricted to the HR repair-pathway, while *BRCA1* is known to have additional functions in DNA repair, involving the *BRCA1*-associated genome-surveillance complex and in transcription-coupled excision repair [36]. The fact that more DNA repair mechanisms are affected by inactivation of *BRCA1* might explain the different molecular subtypes and histology observed in *BRCA1* and *BRCA2* tumors.

The association between the triple-negative/basal-like phenotype and *BRCA1* germline mutation carrier status has been confirmed by several other studies [9], [37]–[39]. However, a minor but still significant fraction of *BRCA1* tumors are ER+ [9], [40]–[42]. A possible explanation could be that ER+ breast cancers in *BRCA1* carriers may be incident and sporadic in nature (phenocopies) and not directly caused by the *BRCA1* inactivation. Several studies have shown a clear association between older age and development of ER+ breast cancers in *BRCA1* mutation carriers [9], [43], [44]. As the same trend is seen in the general population, this could support the hypothesis that the majority of ER+ *BRCA1* breast cancers are just incidental. However, a recent study by Tung et al. indicated that ER+ *BRCA1* breast cancers are different from sporadic ER+ breast cancers matched for age, being more frequently ductal carcinomas with a higher mitotic rate and with the absence of lymphocytic infiltration [42]. In our tumor material, 14 out of 33 *BRCA1* tumors were ER+. Interestingly, the lumB subtype was overrepresented among the ER+ *BRCA1* tumors (9/14). The 9 lumB tumors represents only 8 breast cancer patients, as 2 of the tumors originated from the same individual (carrying a deletion of exon 17–19) with bilateral breast cancer. Among the lumB *BRCA1* carriers, only 3 of 8 (38%) women were diagnosed before the age of 50 years, compared with 14 of 20 (70%) of the basal-like *BRCA1* carriers. As lumB tumors are characterized by a high mitotic index, this is in line with the observations by Tung et al. It has been speculated that ER+ *BRCA1* tumors arise due to haploinsufficiency (with no loss-of-heterozygosity, LOH), arise from a different cell population, are a result of menopause-related metabolic changes, or may be related to genetic differences either by distinct mutations within the *BRCA1* gene or by modifying genes variants [42], [45]. The latter was supported by a recent association study where association of ER+ *BRCA1* breast cancer to a common nucleotide variant in *FGFR2* was found [46].

### Classification of *BRCA1* and *BRCA2*

To investigate whether gene-expression profiles could be used to distinguish tumors from *BRCA1* and *BRCA2* mutation carriers from sporadic cancers, we applied the SVM classification algorithm. The *BRCA1* and *BRCA2* sample sizes were too small for subgrouping into training and test sets, so instead we utilized the LOOCV method to evaluate the classification performance. Our initial approach was to perform a general *BRCA1*-*versus*-sporadic and a *BRCA2*-*versus*-sporadic classification approach without taking any clinically relevant subgrouping into account. *BRCA1*-*versus*-sporadic classification resulted in a balanced accuracy of 77%, but the classification was highly confounded by the molecular subtype. Thorough review of the general *BRCA1* classification, results revealed that all except one basal-like tumor, including all sporadic tumors, were classified as *BRCA1*. Within the group of lumB samples, 6 out of 9 tumors were misclassified. Because of the unequal distributions of subtypes within the *BRCA1* and sporadic groups, the general *BRCA1*-*versus*-sporadic classification mainly distinguished basal-like from non-basal-like tumors. The general *BRCA2*-*versus*-sporadic classification resulted in 83% balanced accuracy. In contrast to the BRCA1 classification, less confounding was observed here. This can either be explained by a more distinct phenotype of *BRCA2* tumors or by the more comparable subtype distributions between *BRCA2* and sporadic samples. The classification performances of the general BRCA1/2 classifiers were compared to what could be achieved by the standard clinical variables: ER, PR, HER2, and age of onset (Table S13). ER and PR status were able to distinguish *BRCA1* from sporadic tumors with 71% and 70% balanced accuracy, respectively, compared to 77% by the general gene-expression based BRCA1-*versus*-sporadic classification. Even though it was found to be highly influenced by molecular tumor subtypes (which are highly correlated with hormone receptor expression), the gene-expression-based classification seemed to be able to capture additional hormone-receptor-independent–*BRCA1*-related biological information. *BRCA2* classification by ER, PR, and HER2 status resulted only in near-random prediction accuracies. Early onset of disease (≤50 years) was able to predict *BRCA1* and *BRCA2* status with 72% and 79% accuracies, respectively. These age-derived estimates are however most likely to be overly optimistic as a consequence of the study design. Because of the very low prevalence of *BRCA1/2* germline mutation carriers among unselected breast cancer patients, the study was designed as a case-cohort study in order to acquire a reasonable number of *BRCA1/2* tumors [47]. The group of *BRCA1/2* mutation carriers therefore represents a highly selected group of patients, where early age of onset in combination with a strong family history have been used to qualify and select patients for BRCA1/2 mutation testing. The group of mutations carriers in the present study is therefore most likely to represent an enriched group of early-onset BRCA1/2 patients. In previous studies of unselected breast cancer cases only 3 to 10% of patients diagnosed at less than age 45 years were reported to carry a *BRCA1/2* mutation [47], [48].

To avoid potential confounding effects related to tumor subtypes, we stratified the tumor samples according to molecular subtype prior to classification. By conducting *BRCA1*-*versus*-sporadic classification within only basal-like samples, we w found that basal-like *BRCA1* tumors could successfully be distinguished from sporadic tumors of the same subtype with high accuracy (balanced accuracy: 83%, sensitivity: 85%, specificity: 80%). Even though these numbers are low estimates, it cannot be excluded that actual sporadic tumors arise in germline *BRCA1* mutation carriers and hence are not caused by germline mutations. Neither can it be excluded that some of the patients in the sporadic group in fact carried a *BRCA1* germline mutation and were therefore not misclassified, as they were not tested for *BRCA1/2* mutations. It could even be argued that by only selecting basal-like tumors, we enriched for tumors from unrecognized *BRCA1* carriers. Due to ethical concerns, permission to do *BRCA1/2* gene testing on the unselected sporadic group was not given, which represents a limitation of the study. A recent study reported a 16% prevalence rate of germline *BRCA1* mutations among unselected triple-negative breast cancers [49]. Although we cannot rule out that a minor fraction of the tumors harbor a *BRCA1* mutation, it seems most likely that the majority of tumors are truly sporadic/non-hereditary in origin. Another consideration was whether the difference in age between the basal-like *BRCA1* group (median age: 41 years, range 25–61) and basal-like sporadic group (median age: 72 years, range 53–87) had had a confounding effect on classification. All 6 “late-onset” (>50 years) *BRCA1* samples was however correctly classified while the 3 misclassified *BRCA1* samples were all early-onset cancers (40–42 years). This indicates that the classification was independent of the age of onset. Low tumor-cell percentage and tumor heterogeneity are other parameters that could have influenced the classifications, though measures had been taken to only include samples with high tumor content.

The less successful *BRCA1*-*versus*-sporadic lumB classification (balanced accuracy: 62%, sensitivity: 44%, specificity: 79%) could indicate that the lumB *BRCA1* tumors were more similar to sporadic tumors. This could be explained by a high number of actual sporadic tumors within the lumB *BRCA1* mutation carrier group due to baseline sporadic risk not related to the *BRCA1* germline mutation. The large difference in group sizes could also have affected the classification, as unbalanced group sizes is well known to have a negative influence on performances of machine-learning algorithms including SVM.

In the lumB-*BRCA2*-*versus*-sporadic classification we observed a minor improvement in prediction accuracy from 83% to 89%. Also here, the classification appeared unaffected by the age differences found between the lumB *BRCA2* samples (median 42 years, range 28–72) and the lumB sporadic samples (64.5 years, range 36–89). For comparison, the performance of age as a predictive variable was evaluated (Table S13). Early-onset of disease (≤50 years) was able to predict *BRCA1* status within basal-like samples and *BRCA2* status within lumB samples with 85% and 75% balanced accuracy, respectively. As discussed previously, the group of *BRCA1/2* mutations carriers in the present study is, however, most likely to represent an enriched group of early-onset *BRCA1/2* patients, as they represent a highly selective group. As a consequence, prediction estimates using age of onset in our study group are likely to be misleading. In support, the recent study from the Hellenic Cooperative Oncology Group found that only 27% of women with triple-negative breast cancer, unselected for family history, had a *BRCA1* mutation [49]. Although, early onset of disease is usable as selection criteria for *BRCA1* genetic testing among triple-negative breast cancer patients, age alone cannot predict *BRCA1* involvement.

The classification results indicate that *BRCA1* tumors and *BRCA2* tumors represent distinct biological entities among basal-like and lumB tumors, respectively. This is supported by recent studies showing that specific copy number aberrations differed between *BRCA1/2* and sporadic tumors [14], [25], [26], [50]. Only 3 studies have investigated the classification potential of gene-expression tumor profiles in relation to *BRCA1/2* mutation status. Hedenfalk et al. was able to distinguish *BRCA1* from non-*BRCA1* samples with high accuracy; however concerns have been raised because of small sample sizes and a lack of appropriate matching according to clinical parameters such as ER status. Their prediction of *BRCA2* mutation carrier status was less accurate [18], [24]. In studies by van't Veer et al. and Lisowska et al., samples were matched according to ER status (but not HER2 status) prior to *BRCA1* classification [19], [20]. Lisowska obtained only near-random classification while van't Veer achieved high accuracies. But even though LOOCV was used to assess classification performance the result may be biased due to possible information-leakage, as selection of classifier-genes involved usages of the complete set of samples.

For validation purposes, we then developed a 110-gene basal *BRCA1* signature and a 100-gene lumB *BRCA2* signature. The genes *KIAA0100* and *RPL23A*, both contained in the *BRCA1* signature, were also found in the *BRCA1* reporter gene list reported by van't Veer et al. [19]. Interestingly, both genes are located at 17q11.2 and show lowest expression in *BRCA1* tumors, which could indicate that loss of this region may be associated with the development of the basal-like *BRCA1* tumor type.

To investigate whether the gene-expression patterns of the *BRCA1/2* signatures were reproducible we performed a technical validation by analyzing the same samples using another microarray platform. We chose the cross-platform-validation analysis rather than the traditional qPCR validation often utilized in gene-expression studies, as it provided a high degree of flexibility and was readily available in our laboratory. It should be emphasized that the cross-platform classification is to be considered as training results as the tested samples were used to develop the signatures. Nevertheless, the results indicated high reproducibility of the gene-expression measurement of the signatures across the two microarray platforms.

Finally, and most importantly, we sought to validate our *BRCA1/2* signatures in a set of independent samples. None of the previously published *BRCA1/2* signatures have ever been externally validated. Suitable for validation purposes, we were able to identify two publicly available gene expression data sets. Interestingly, using the two independent data sets, we were able to successfully validate both the *BRCA1* and *BRCA2* signature with high accuracies (82–87%). Our results support the hypothesis that *BRCA1*-associated tumors represent a distinct biological subgroup among basal-like tumors, which have been a topic of debate. Likewise, *BRCA2-*associated tumors pose a distinct subgroup among lumB tumors.

## Conclusions

We have developed and validated subtype-specific gene signatures and demonstrated that they can be used to predict *BRCA1* association among basal-like tumors and *BRCA2* association among lumB tumors with high accuracies. To the best of our knowledge, this is the first study to validate *BRCA1/2* gene-expression signatures in independent external data sets. Although additional validation studies are required, microarray gene-expression analysis on fresh/frozen tissues, utilizing our *BRCA1/2* signatures in combination with PAM50 subtype classification, could potentially be valuable as a functional assay in genetic diagnostics to identify *BRCA1/2* involvement (BRCAness). Furthermore, transferring the signatures to a PCR-based platform or analyzing the signatures by target RNA sequencing using next-generation sequencing would enable analysis of RNA from Formalin-Fixed, Paraffin-Embedded (FFPE) tissue. Used as a functional assay, it could help facilitate the clinical interpretation of the large number of sequence variants of unknown clinical significance found in the *BRCA1/2* genes for distinguishing pathogenic mutations from benign variants. Potentially, the signatures could also be used as a tool for preselecting patients for mutation screening, as a significant proportion of *BRCA1* and *BRCA2* germline mutation carriers do not have a family history of breast cancers. New targeted therapies such as PARP inhibitors have been demonstrated to be effective treatments for *BRCA1/2* mutation carriers due to dysfunctional HR DNA repair. In addition to germline mutations, other mechanisms, such as somatic and epigenetic inactivation of *BRCA1/2*, can lead to *BRCA*-deficiency and impaired HR DNA repair. Finally, our gene signatures could potentially prove to provide a general method for detecting *BRCA*-deficient tumors sensitive to new targets therapies making it applicable for optimal treatment decisions.

## Supporting Information

Methods S1 **Preparation of validation datasets.**
(PDF)

Figure S1 **Derivation of ER, PR and HER2 status from gene expression data.** Gene expression measurements of ESR1, PGR and ERBB2 were used to determine ER, PR and HER2 status, respectively. Receiver operating characteristic (ROC) curves generated from samples with available immunohistochemical data showed large areas under curves (AUC), indicating high discriminatory power of the gene expression measurements. Density plots revealed bimodal distributions of ESR1, PGR and ERBB2 expression across all samples and were used to determine arbitrary expression cut-offs (marked as dashed lines) defining ER, PR, and HER2 status, respectively.
(PDF)

Figure S2 **Optimization of the number of predictive reporter genes to be included in the basal BRCA1 signature (A) and lumB BRCA2 signature (B).** For BRCA1 classification 110 genes were found to be the lowest number of genes providing the highest mean balanced accuracy, while 100 genes were the most optimal for BRCA2 classification. See Materials and methods section for more details.
(PDF)

Figure S3 **PCA plots.** A) Basal-like BRCA1 (n = 20) and sporadic (n = 10) tumors visualized using the 110 reporter genes included in the basal BRCA1 signature. B) Luminal B BRCA2 (n = 16) and sporadic (n = 48) tumors visualized using the 100 reporter genes included in the BRCA2 signature.
(PDF)

Table S1 **BRCA1/2 germline mutations and its relation to molecular tumor subtypes.** Mutations are all known pathogenic mutation described using HGVS nomenclature.
(PDF)

Table S2 **Patient and tumor characteristics of** ***BRCA1*** **mutation carriers in relation to molecular subtypes.**
(PDF)

Table S3 **Patient and tumor characteristics of** ***BRCA2*** **mutation carriers in relation to molecular subtypes.**
(PDF)

Table S4 **Patient and tumor characteristics of sporadic patients in relation to molecular subtypes.**
(PDF)

Table S5 **Evaluation of the general** ***BRCA1*** **and** ***BRCA2*** **classifications within each of the molecular subtype sample groups.**
(PDF)

Table S6 ***BRCA1*** **classification results of basal-like** ***BRCA1*** **(** ***n*** **= 20) and basal-like sporadic (** ***n =*** **10) tumors obtained using leave-one-out cross-validation.** See Materials and methods section for more details. Mutations are all known pathogenic mutation described using HGVS nomenclature.
(PDF)

Table S7 ***BRCA1*** **classification results of lumB** ***BRCA1*** **(** ***n*** **= 9) and lumB sporadic (** ***n =*** **48) tumors obtained using leave-one-out cross-validation.** See Materials and methods section for more details. Mutations are all known pathogenic mutation described using HGVS nomenclature.
(PDF)

Table S8 ***BRCA2*** **classification results of lumB** ***BRCA2*** **(** ***n*** **= 16) and lumB sporadic (** ***n =*** **48) tumors obtained using leave-one-out cross-validation.** See Materials and methods section for more details. Mutations are all known pathogenic mutation described using HGVS nomenclature.
(PDF)

Table S9 **The basal BRCA1 signature.** 76 out of 110 genes were contained on the Rosetta chip used in the NKI study and 69 genes were present in the Jönsson dataset (indicated by ×).
(PDF)

Table S10 **The lumB BRCA2 signature.** 77 out of the 100 genes were present in the Jönsson dataset (indicated by ×).
(PDF)

Table S11 **Distribution of predicted molecular subtypes within the NKI dataset.**
(PDF)

Table S12 **Distribution of predicted molecular subtypes within the Jönsson dataset.**
(PDF)

Table S13 **BRCA1/2 classification results using the standard clinical variables ER, PR, HER2, TNBC (ER−/PR−/HER2−) and age of onset of disease.**
(PDF)
