# Supplementary material for: Classifications within Molecular Subtypes Enables Identification of BRCA1/BRCA2 Mutation Carriers by RNA Tumor Profiling
Source: PLoS One. 2013 May 21;8(5):e64268. doi: 10.1371/journal.pone.0064268 (PMC3660328; doi:10.1371/journal.pone.0064268)
Supplement: Methods S1 — Preparation of validation datasets. (PDF) [file pone.0064268.s001.pdf]

Methods S1

Preparation of validation datasets

In-house spotted microarray analysis

For cross-platform validation of the gene signatures, a subset (20 basal-like BRCA1, 10 basal-like sporadic, 16 lumB BRCA2 and 46 lumB sporadic, in total 92 samples) of the tumor samples analyzed by Agilent SurePrint G3 arrays were also analyzed by our in-house spotted microarray platform. The spotted gene expression microarray platform has previously been validated by our group [1]. For fabrication of the spotted arrays, a human oligonucleotide library consisting of 28,919 DNA oligonucleotide probes (60-mer) was purchased from Compugen-Sigma-Genosys (The Woodlands). The oligonucleotide probes were solubilized in 150 mM sodium phosphate buffer (pH 8.5) to a final concentration of 20 pmol/µl and spotted onto CodeLink HD (SurModics) activated glass slides by a high-precision spotting robot (Virtek ChipWriter Pro, ESI). The slides have an active polymer coating of amine-reactive groups permitting the 5'-amino modified DNA oligos to covalently attach under high relative humidity. The microarray fabrication was carried out in a controlled environment at 38% humidity and the temperature was held constant at 23 °C. SMP 2.5 stealth pins (TeleChem Interational) was used to deposit the oligos onto surface of the slides. Up to 85 arrays were spotted per batch. After printing, slides were incubated overnight at 70% humidity and blocked as recommended by the manufacturer. RNA was amplified and labeled using the Amino Allyl MessageAmp II aRNA Amplification Kit (Ambion) according to the manufacturer's protocol. Amplified aRNA from the tumor samples were labeled with Cy5. Universal Human Reference RNA (Stratagene) was labeled with Cy3 and used as reference RNA. Fragmentation, hybridization and washing for the spotted arrays were carried out using Agilent Gene Expression Hybridization Kit (Agilent Technologies) and Gene Expression Wash Buffer Kit (Agilent Technologies) according to the manufacturer's protocol in a low ozone environment. Subsequently, the arrays were scanned using a Agilent G2565CA Microarray scanner (Agilent Technologies) and the scanned images were quantified by Gene Pix Pro 6.0 (Molecular Devices). The data pre-processing was performed as described for the Agilent SurePrint G3 arrays. The probes were re-annotated to gene symbols by Agilent eArray tool (http://earray.chem.agilent.com/earray) using the original probe sequences provided by the manufacturer. The 92 samples were processed together with 154 other breast tumor samples. ComBat method was used for adjustment of batch effects across different spotting batches [2]. Missing expression values were imputed by *k*-nearest neighbors averaging (k = 10). Microarray data have been deposited into the Gene Expression Omnibus (GSE40115).

Jönsson dataset

The Jönsson dataset was downloaded from NCBI's Gene Expression Omnibus repository (GSE22133) as raw GenePix files [3]. The dataset consisted of 346 primary tumor samples (17 *BRCA1*, 31 *BRCA2*, 126 familial non-*BRCA1/2* and 172 sporadic) and 13 metastatic samples. Only primary tumor samples were included in the following analysis. Flagged features were removed from the analysis and the remaining data were pre-processed as described in the materials and methods section. Briefly, normexp method was used for background correction and the background corrected data were within-array normalized using the loess method and between-array normalized using the quantile method. The probes were mapped to gene symbols from the provided Ensembl Gene ID by DAVID Gene ID Conversion Tool. In cases of multiple probes per gene symbol only the probe with the maximum mean intensity (calculated using Cy3 intensity data) was kept. Classification of the samples into molecular subtypes was performed using the PAM50 classifier as described in the materials and methods section. 43 out of the 50 genes comprising PAM50 could be mapped to a probe in the Jönsson dataset.

NKI dataset

The NKI datset was downloaded from Netherlands Cancer Institute website (http://bioinformatics.nki.nl) [4]. The NKI dataset consisted of 117 primary tumor samples (18 *BRCA1* samples, 2 *BRCA2* and 97 Sporadic). All probes were re-annotated to gene symbols by Agilent eArray tool (http://earray.chem.agilent.com/earray), using the original oligo sequences provided by the authors. In cases of multiple probes per gene symbol only the probe with the highest variance across all samples was kept. Classification of the samples into molecular subtypes was performed using the PAM50 classifier as described in the materials and methods section. 49 out of the 50 genes comprising PAM50 could be mapped to a probe in the NKI dataset.

References

1. Thomassen M, Skov V, Eiriksdottir F, Tan Q, Jochumsen K, Fritzner N, Brusgaard K, Dahlgaard J, Kruse TA: **Spotting and validation of a genome wide oligonucleotide chip with duplicate measurement of each gene**. *Biochem Biophys Res Commun* 2006, **344**:1111–20.

2. Johnson WE, Li C, Rabinovic A: **Adjusting batch effects in microarray expression data using empirical Bayes methods**. *Biostatistics* 2007, **8**:118–127.

3. Jönsson G, Staaf J, Vallon-Christersson J, Ringnér M, Holm K, Hegardt C, Gunnarsson H, Fagerholm R, Strand C, Agnarsson BA, Kilpivaara O, Luts L, Heikkilä P, Aittomäki K, Blomqvist C, Loman N, Malmström P, Olsson H, Th Johannsson O, Arason A, Nevanlinna H, Barkardottir RB, Borg Å: **Genomic subtypes of breast cancer identified by array-comparative genomic hybridization display distinct molecular and clinical characteristics**. *Breast Cancer Research* 2010, **12**:R42.

4. van 't Veer LJ, Dai H, van de Vijver MJ, He YD, Hart AAM, Mao M, Peterse HL, van der Kooy K, Marton MJ, Witteveen AT, Schreiber GJ, Kerkhoven RM, Roberts C, Linsley PS, Bernards R, Friend SH: **Gene expression profiling predicts clinical outcome of breast cancer**. *Nature* 2002, **415**:530–6.
