# Supplementary material for: Classifications within Molecular Subtypes Enables Identification of BRCA1/BRCA2 Mutation Carriers by RNA Tumor Profiling
Source: PLoS One. 2013 May 21;8(5):e64268. doi: 10.1371/journal.pone.0064268 (PMC3660328; doi:10.1371/journal.pone.0064268)
Supplement: Figure S1 — Derivation of ER, PR and HER2 status from gene expression data. Gene expression measurements of ESR1, PGR and ERBB2 were used to determine ER, PR and HER2 status, respectively. Receiver operating characteristic (ROC) curves generated from samples with available immunohistochemical data showed large areas under curves (AUC), indicating high discriminatory power of the gene expression measurements. Density plots revealed bimodal distributions of ESR1, PGR and ERBB2 expression across all samples and were used to determine arbitrary expression cut-offs (marked as dashed lines) defining ER, PR, and HER2 status, respectively. (PDF) [file pone.0064268.s002.pdf]

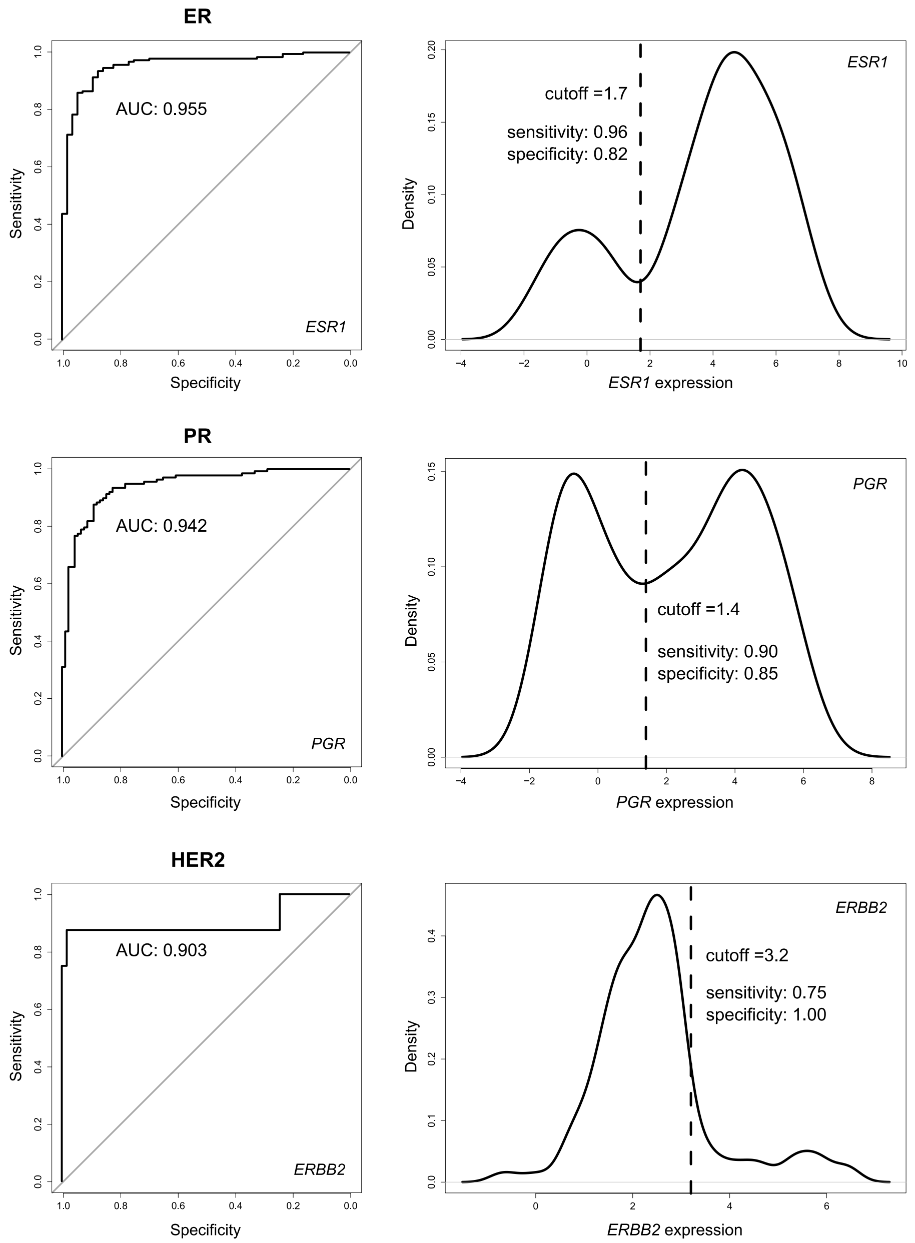

Figure S1. Derivation of ER, PR and HER2 status from gene expression data. Gene expression measurements of *ESR1*, *PGR* and *ERBB2* were used to determine ER, PR and HER2 status, respectively. Receiver operating characteristic (ROC) curves generated from samples with available immunohistochemical data showed large areas under curves (AUC), indicating high discriminatory power of the gene expression measurements. Density plots revealed bimodal distributions of *ESR1*, *PGR* and *ERBB2* expression across all samples and were used to determine arbitrary expression cut-offs (marked as dashed lines) defining ER, PR, and HER2 status, respectively.
