# Supplementary material for: Classifications within Molecular Subtypes Enables Identification of BRCA1/BRCA2 Mutation Carriers by RNA Tumor Profiling
Source: PLoS One. 2013 May 21;8(5):e64268. doi: 10.1371/journal.pone.0064268 (PMC3660328; doi:10.1371/journal.pone.0064268)
Supplement: Figure S2 — Optimization of the number of predictive reporter genes to be included in the basal BRCA1 signature (A) and lumB BRCA2 signature (B). For BRCA1 classification 110 genes were found to be the lowest number of genes providing the highest mean balanced accuracy, while 100 genes were the most optimal for BRCA2 classification. See Materials and methods section for more details. (PDF) [file pone.0064268.s003.pdf]

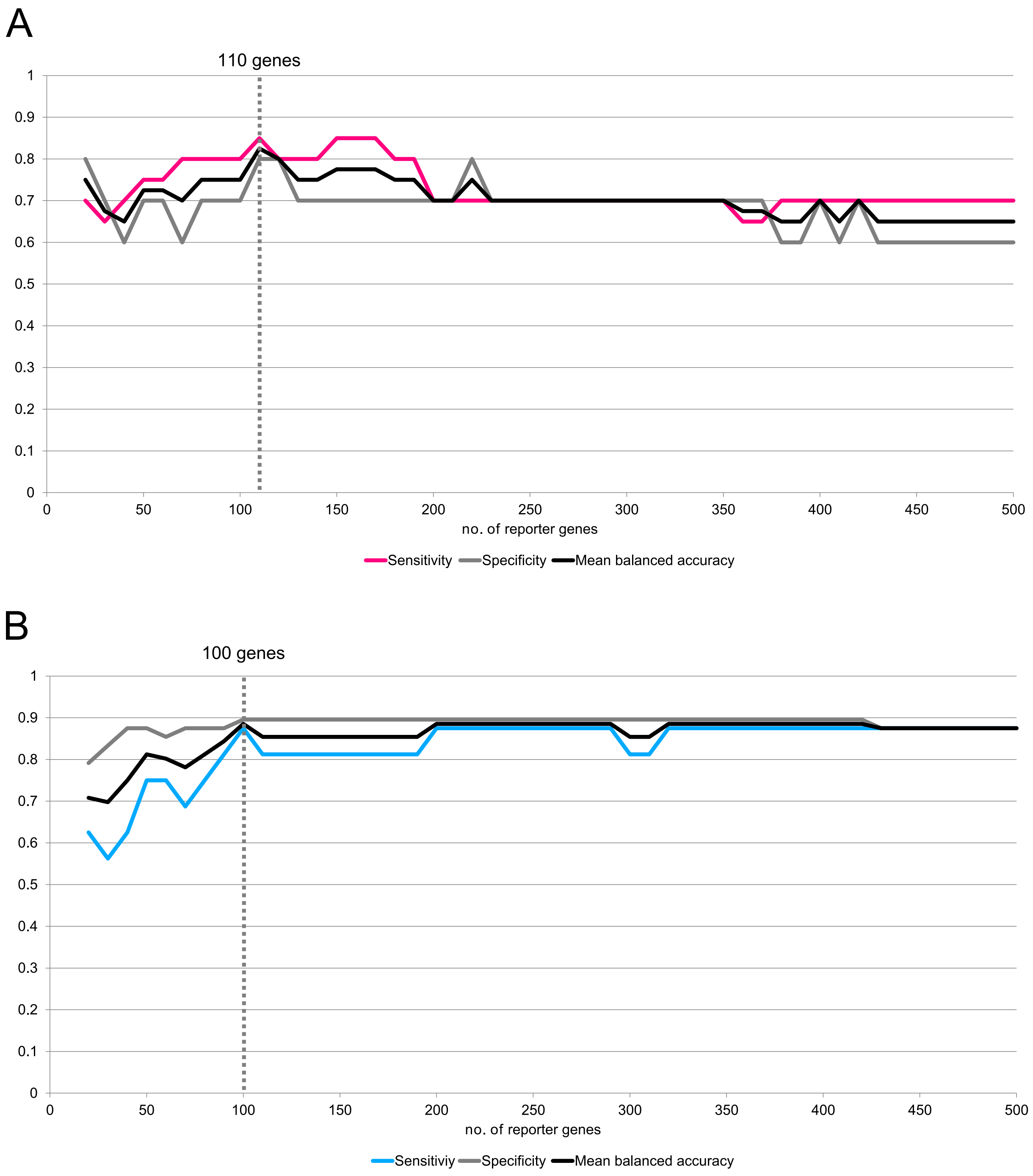

Figure S2. Optimization of the number of predictive reporter genes to be included in the basal *BRCA1* signature (A) and lumB *BRCA2* signature (B). For *BRCA1* classification 110 genes were found to be the lowest number of genes providing the highest mean balanced accuracy, while 100 genes were the most optimal for *BRCA2* classification. See Materials and methods section for more details.
