# Supplementary material for: Classifications within Molecular Subtypes Enables Identification of BRCA1/BRCA2 Mutation Carriers by RNA Tumor Profiling
Source: PLoS One. 2013 May 21;8(5):e64268. doi: 10.1371/journal.pone.0064268 (PMC3660328; doi:10.1371/journal.pone.0064268)
Supplement: Figure S3 — PCA plots. A) Basal-like BRCA1 (n = 20) and sporadic (n = 10) tumors visualized using the 110 reporter genes included in the basal BRCA1 signature. B) Luminal B BRCA2 (n = 16) and sporadic (n = 48) tumors visualized using the 100 reporter genes included in the BRCA2 signature. (PDF) [file pone.0064268.s004.pdf]

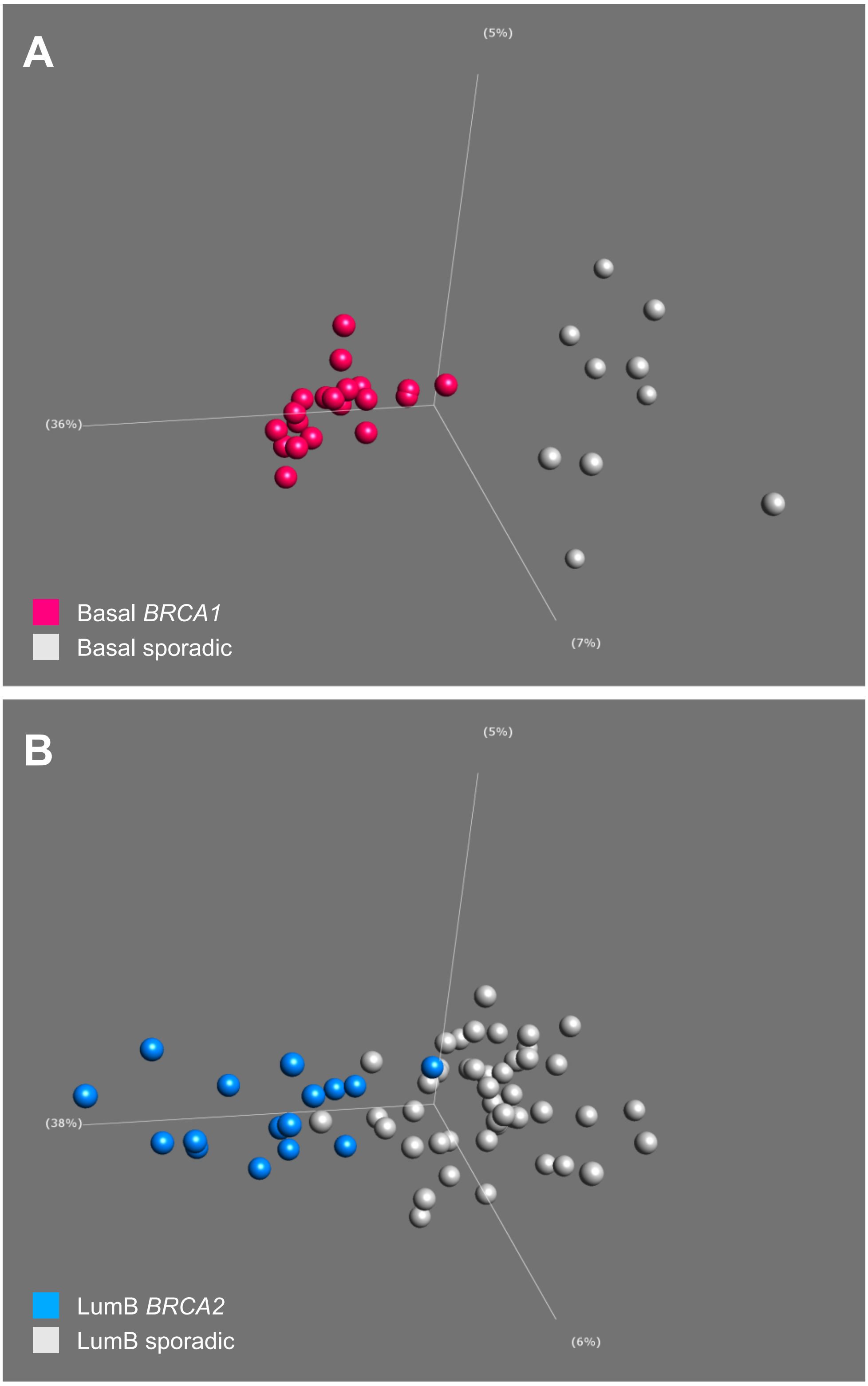

Figure S3. PCA plots. A) Basal-like *BRCA1* (*n* = 20) and sporadic (*n* = 10) tumors visualized using the 110 reporter genes included in the basal *BRCA1* signature. B) Luminal B *BRCA2* (*n* = 16) and sporadic (*n* = 48) tumors visualized using the 100 reporter genes included in the *BRCA2* signature.
