# Supplementary material for: Classifications within Molecular Subtypes Enables Identification of BRCA1/BRCA2 Mutation Carriers by RNA Tumor Profiling
Source: PLoS One. 2013 May 21;8(5):e64268. doi: 10.1371/journal.pone.0064268 (PMC3660328; doi:10.1371/journal.pone.0064268)
Supplement: Table S1 — BRCA1/2 germline mutations and its relation to molecular tumor subtypes. Mutations are all known pathogenic mutation described using HGVS nomenclature. (PDF) [file pone.0064268.s005.pdf]

Table S1. *BRCA1/2* germline mutations and its relation to molecular tumor subtypes. Mutations are all known pathogenic mutation described using HGVS nomenclature.

	Basal	HER2	LumA	LumB	Normal	Functional effects
BRCA1						
c.115T>G, Exon3, p.(Cys39Gly)	-	-	-	1	-	MS
c.181T>G, Exon5, p.(Cys61Gly)	2	-	-	-	1	MS
c.1082_1092del, Exon11, p.(Ser361*)	-	-	1	-	-	NS
c.1505delT, Exon11, p.(Leu502*)	1	-	-	-	-	FS
c.1556delA, Exon11, p.(Lys519Argfs*13)	1	-	-	-	-	FS
c.2475delC, Exon11, p.(Asp825Glufs*21)	1	-	-	3	-	FS
c.2476delA, Exon11, p.(Thr826Glnfs*20)	1	-	-	1	-	FS
c.3319G>T, Exon11, p.(Glu1107*)	2	-	-	-	-	NS
c.3400G>T, Exon11, p.(Glu1134*)	1	1	-	-	-	NS
c.4389C>A, Exon14, p.(Tyr1463*)	-	1	-	-	-	NS
c.5089T>C, Exon18, p.(Cys1697Arg)	3	-	-	-	-	MS
c.5143A>C, Exon18, p.(Ser1715Arg)	1	-	-	-	-	MS
c.5213G>A, Exon20, p.(Gly1738Glu)	3	-	-	-	-	MS
c.5266dupC, Exon20, p.(Gln1756Profs*74)	3	-	-	1	-	FS
c.5503C>T, Exon24, p.(Arg1835*)	-	-	-	1	-	FS
c.5153-?_5193+?del, Exon19, p.(Trp1718Serfs*2)	1	-	-	-	-	Exon del. (FS)
c.4987-?_5193+?del, Exon17-19, p.(Met1663_Glu1731del)	-	-	-	2	-	Exon del. (IF)
BRCA2						
c.145G>T, Exon3, p.(Glu49*)	-	-	-	-	1	NS
c.1310_1313delAAGA, Exon10, p.(Lys437Ilefs*22)	-	-	-	2	-	FS
c.1813delA, Exon10, p.(Ile605Tyrfs*9)	1	-	-	-	-	FS
c.2808_2811delACAA, Exon11, p.(Ala938Profs*21)	-	-	-	2	-	FS
c.2830A>T, Exon11, p.(Lys944*)	-	-	-	3	-	NS
c.3530_3533delACAG, Exon11, p.(Asp1177Alafs*19)	-	-	-	1	-	FS
c.5576_5579delTTAA, Exon11, p.(Ile1859Lysfs*3)	-	-	1	-	-	FS
c.5754delT, Exon11, p.(His1918Glnfs*45)	1	-	-	-	-	FS
c.6373delA, Exon11, p.(Thr2125Profs*12)	-	-	-	1	-	FS
c.6486_6489delACAA, Exon11, p.(Lys2162Asnfs*5)	-	-	1	2	-	FS
c.6490_6492delinsGACT, Exon11, p.(Gln2164Aspfs*12)	-	-	-	1	-	FS
c.6601delT, Exon11, p.(Ser2201Leufs*5)	-	-	-	1	-	FS
c.8575delC, Exon20, p.(Gln2859Lysfs*4)	-	-	1	1	-	FS
c.9015delA, Exon23, p.(Arg3005Serfs*23)	-	-	-	1	-	FS
c.7617+1G>A, Splice mut, Exon15 skipping	-	-	-	1	-	Exon skipping

Abbreviations: MS, missense mutation; FS, frameshift mutation; NS, nonsense mutation; IF, in-frame mutation
