# Supplementary material for: Classifications within Molecular Subtypes Enables Identification of BRCA1/BRCA2 Mutation Carriers by RNA Tumor Profiling
Source: PLoS One. 2013 May 21;8(5):e64268. doi: 10.1371/journal.pone.0064268 (PMC3660328; doi:10.1371/journal.pone.0064268)
Supplement: Table S2 — Patient and tumor characteristics of BRCA1 mutation carriers in relation to molecular subtypes. (PDF) [file pone.0064268.s006.pdf]

Table S2. Patient and tumor characteristics of *BRCA1* mutation carriers in relation to molecular subtypes

***BRCA1* tumors**	**Basal (*n* = 20)**	**HER2 (*n* = 2)**	**LumA (*n* = 1)**	**LumB (*n* = 9)**	**Normal (*n* = 1)**
**Estrogen receptor**					
ER+	2	2	1	9	0
ER-	18	0	0	0	1
**Progesterone receptor**					
PR+	0	2	1	3	1
PR-	20	0	0	6	0
**HER2 status**					
HER2+	0	2	0	1	0
HER2-	20	0	1	8	1
**Histologic grade**					
Grade 1	0	0	0	3	0
Grade 2	4	0	1	1	1
Grade 3	12	2	0	4	0
NA	4	0	0	1	0
**Tumor type**					
Invasive ductal carcinoma	16	2	1	8	1
Invasive lobular carcinoma	1	0	0	0	0
Mucinous carcinoma	0	0	0	0	0
Medullary carcinoma	2	0	0	0	0
Tubular carcinoma	0	0	0	0	0
Metaplastic carcinoma	0	0	0	0	0
Other	0	0	0	0	0
NA	1	0	0	1	0
**Age**					
< 50 years	14	2	1	3	1
≥ 50 years	6	0	0	6	0
**Menupause status**					
Premenopausal	14	2	1	2	1
Perimenopausal	0	0	0	0	0
Postmenopausal	5	0	0	7	0
Other	0	0	0	0	0
NA	0	0	0	0	0
