# Supplementary material for: Classifications within Molecular Subtypes Enables Identification of BRCA1/BRCA2 Mutation Carriers by RNA Tumor Profiling
Source: PLoS One. 2013 May 21;8(5):e64268. doi: 10.1371/journal.pone.0064268 (PMC3660328; doi:10.1371/journal.pone.0064268)
Supplement: Table S3 — Patient and tumor characteristics of BRCA2 mutation carriers in relation to molecular subtypes. (PDF) [file pone.0064268.s007.pdf]

Table S3. Patient and tumor characteristics of *BRCA2* mutation carriers in relation to molecular subtypes

BRCA2 tumors	Basal (*n* = 2)	HER2 (*n* = 0)	LumA (*n* = 3)	LumB (*n* = 16)	Normal (*n* = 1)
**Estrogen receptor**					
ER+	0	0	3	16	1
ER-	2	0	0	0	0
**Progesterone receptor**					
PR+	0	0	3	12	1
PR-	2	0	4	0	0
**HER2 status**					
HER2+	0	0	0	1	0
HER2-	2	0	3	15	1
**Histologic grade**					
Grade 1	0	0	1	1	0
Grade 2	2	0	1	8	0
Grade 3	0	0	0	6	1
NA	0	0	1	1	0
**Tumor type**					
Invasive ductal carcinoma	2	0	2	15	0
Invasive lobular carcinoma	0	0	1	0	1
Mucinous carcinoma	0	0	0	0	0
Medullary carcinoma	0	0	0	0	0
Tubular carcinoma	0	0	0	0	0
Metaplastic carcinoma	0	0	0	0	0
Other	0	0	0	0	0
NA	0	0	0	1	0
**Age**					
< 50 years	1	0	1	12	1
≥ 50 years	1	0	2	4	0
**Menupause status**					
Premenopausal	1	0	2	11	1
Perimenopausal	0	0	0	1	0
Postmenopausal	1	0	1	3	0
Other	0	0	0	0	0
NA	0	0	0	1	0
