# Supplementary material for: Classifications within Molecular Subtypes Enables Identification of BRCA1/BRCA2 Mutation Carriers by RNA Tumor Profiling
Source: PLoS One. 2013 May 21;8(5):e64268. doi: 10.1371/journal.pone.0064268 (PMC3660328; doi:10.1371/journal.pone.0064268)
Supplement: Table S4 — Patient and tumor characteristics of sporadic patients in relation to molecular subtypes. (PDF) [file pone.0064268.s008.pdf]

Table S4. Patient and tumor characteristics of sporadic patients in relation to molecular subtypes

Sporadic tumors	Basal (n = 10)	HER2 (n = 14)	LumA (n = 55)	LumB (n = 48)	Normal (n = 1)
**Estrogen receptor**					
ER+	0	3	55	48	1
ER-	10	11	0	0	0
**Progesterone receptor**					
PR+	0	1	47	30	1
PR-	10	13	8	18	0
**HER2 status**					
HER2+	1	13	4	3	0
HER2-	9	1	51	45	1
**Histologic grade**					
Grade 1	1	0	27	4	0
Grade 2	0	4	19	25	0
Grade 3	8	9	0	11	1
NA	1	1	9	8	0
**Tumor type**					
Invasive ductal carcinoma	10	13	42	39	1
Invasive lobular carcinoma	0	0	9	3	0
Mucinous carcinoma	0	0	1	1	0
Medullary carcinoma	0	0	0	1	0
Tubular carcinoma	0	0	2	1	0
Metaplastic carcinoma	0	0	0	0	0
Other	0	0	0	2	0
NA	0	1	1	1	0
**Age**					
< 50 years	0	3	8	9	1
≥ 50 years	10	11	47	39	0
**Menupause status**					
Premenopausal	1	3	12	13	1
Perimenopausal	2	2	4	7	0
Postmenopausal	6	8	37	27	0
Other	0	0	2	0	0
NA	1	1	0	1	0
