# Supplementary material for: Classifications within Molecular Subtypes Enables Identification of BRCA1/BRCA2 Mutation Carriers by RNA Tumor Profiling
Source: PLoS One. 2013 May 21;8(5):e64268. doi: 10.1371/journal.pone.0064268 (PMC3660328; doi:10.1371/journal.pone.0064268)
Supplement: Table S5 — Evaluation of the general BRCA1 and BRCA2 classifications within each of the molecular subtype sample groups. (PDF) [file pone.0064268.s009.pdf]

Table S5. Evaluation of the general *BRCA1* and *BRCA2* classifications within each of the molecular subtype sample groups

	No. of samples (*BRCA1* / sporadic)	Sensitivity (TP)	Specificity (TN)	Accuracy[a]	*p*-value[b]
***BRCA1* classification**					
Basal-like	20 / 10	0.95 (19)	0.00 (0)	0.48	1.00
HER2-enriched	2 / 14	0.00 (0)	0.79 (11)	0.39	1.00
Luminal A	1 / 55	1.00 (1)	0.96 (53)	0.98	0.05
Luminal B	9 / 48	0.33 (3)	0.92 (44)	0.63	0.07
Normal-like	1 / 1	0.00 (0)	1.00 (1)	0.50	1.00

	No. of samples (*BRCA2* / sporadic)	Sensitivity (TP)	Specificity (TN)	Accuracy[a]	*p*-value[b]
***BRCA2* classification**					
Basal-like	2 / 10	1.00 (2)	0.90 (9)	0.95	0.04
HER2-enriched	0 / 14	-	0.79 (11)	-	-
Luminal A	3 / 55	0.33 (1)	0.89 (49)	0.61	0.33
Luminal B	16 / 48	0.88 (14)	0.81 (39)	0.84	1.4×10^{-6}
Normal-like	1 / 1	1.00 (1)	1.00 (1)	1.00	1.00

[a] Mean balanced accuracy

[b] Fisher's exact test

Abbreviations: TP, true positive; TN, true negative
