# Supplementary material for: Classifications within Molecular Subtypes Enables Identification of BRCA1/BRCA2 Mutation Carriers by RNA Tumor Profiling
Source: PLoS One. 2013 May 21;8(5):e64268. doi: 10.1371/journal.pone.0064268 (PMC3660328; doi:10.1371/journal.pone.0064268)
Supplement: Table S6 — BRCA1 classification results of basal-like BRCA1 ( n = 20) and basal-like sporadic ( n = 10) tumors obtained using leave-one-out cross-validation. See Materials and methods section for more details. Mutations are all known pathogenic mutation described using HGVS nomenclature. (PDF) [file pone.0064268.s010.pdf]

Table S6. *BRCA1* classification results of basal-like *BRCA1* (*n* = 20) and basal-like sporadic (*n* = 10) tumors obtained using leave-one-out cross-validation. See Materials and methods section for more details. Mutations are all known pathogenic mutation described using HGVS nomenclature.

SampleID	Group	Age	Prediction	Type	Grade	Mutation	Functional effects
A018	*BRCA1*	25	*BRCA1*	IDC	2	*BRCA1* c.181T>G, Exon5, p.(Cys61Gly)	MS
A136	*BRCA1*	31	*BRCA1*	IDC	3	*BRCA1* c.5266dupC, Exon20, p.(Gln1756Profs*74)	FS
A089	*BRCA1*	32	*BRCA1*	IDC	3	*BRCA1* c.181T>G, Exon5, p.(Cys61Gly)	MS
A140	*BRCA1*	32	*BRCA1*	ILC	NA	*BRCA1* c.1505delT, Exon11, p.(Leu502*)	NS
A122	*BRCA1*	36	*BRCA1*	IDC	3	*BRCA1* c.3319G>T, Exon11, p.(Glu1107*)	NS
A002	*BRCA1*	36	*BRCA1*	IDC	3	*BRCA1* c.1556delA, Exon11, p.(Lys519Argfs*13)	FS
A017	*BRCA1*	37	*BRCA1*	NA	NA	*BRCA1* c.2476delA, Exon11, p.(Thr826Glnfs*20)	FS
A134	*BRCA1*	38	*BRCA1*	IDC	3	*BRCA1* c.5266dupC, Exon20, p.(Gln1756Profs*74)	FS
A014	*BRCA1*	40	Sporadic	IDC	3	*BRCA1* c.5266dupC, Exon20, p.(Gln1756Profs*74)	FS
A087	*BRCA1*	41	*BRCA1*	IDC	3	*BRCA1* c.5089T>C, Exon18, p.(Cys1697Arg)	MS
A142	*BRCA1*	41	Sporadic	IDC	2	*BRCA1* c.5089T>C, Exon18, p.(Cys1697Arg)	MS
A112	*BRCA1*	42	Sporadic	IDC	3	*BRCA1* c.5213G>A, Exon20, p.(Gly1738Glu)	MS
A156	*BRCA1*	45	*BRCA1*	IDC	3	*BRCA1* c.3319G>T, Exon11, p.(Glu1107*)	NS
A015	*BRCA1*	46	*BRCA1*	Medullary	NA	*BRCA1* c.5213G>A, Exon20, p.(Gly1738Glu)	MS
A006	*BRCA1*	52	*BRCA1*	IDC	3	*BRCA1* c.5089T>C, Exon18, p.(Cys1697Arg)	MS
A051	*BRCA1*	55	*BRCA1*	IDC	3	*BRCA1* c.5143A>C, Exon18, p.(Ser1715Arg)	MS
A138	*BRCA1*	55	*BRCA1*	IDC	3	*BRCA1* c.2475delC, Exon11, p.(Asp825Glufs*21)	FS
A126	*BRCA1*	56	*BRCA1*	IDC	2	*BRCA1* c.5213G>A, Exon20, p.(Gly1738Glu)	MS
A145	*BRCA1*	60	*BRCA1*	IDC	2	*BRCA1* c.5153-?_5193+?del, Exon19, p.(Trp1718Serfs*2)	Exon del (FS)
A131	*BRCA1*	61	*BRCA1*	Medullary	NA	*BRCA1* c.3400G>T, Exon11, p.(Glu1134*)	NS
K005	Sporadic	53	Sporadic	IDC	3		
K085	Sporadic	55	Sporadic	IDC	3		
K018	Sporadic	60	*BRCA1*	IDC	3		
K073	Sporadic	66	Sporadic	IDC	3		
K170	Sporadic	69	Sporadic	IDC	3		
K053	Sporadic	75	Sporadic	IDC	3		
K041	Sporadic	75	*BRCA1*	IDC	3		
K145	Sporadic	76	Sporadic	IDC	NA		
K001	Sporadic	76	Sporadic	IDC	3		
K177	Sporadic	87	Sporadic	IDC	1		

Abbreviations: MS, missense mutation; FS, frameshift mutation; NS, nonsense mutation
