# Supplementary material for: Classifications within Molecular Subtypes Enables Identification of BRCA1/BRCA2 Mutation Carriers by RNA Tumor Profiling
Source: PLoS One. 2013 May 21;8(5):e64268. doi: 10.1371/journal.pone.0064268 (PMC3660328; doi:10.1371/journal.pone.0064268)
Supplement: Table S7 — BRCA1 classification results of lumB BRCA1 ( n = 9) and lumB sporadic ( n = 48) tumors obtained using leave-one-out cross-validation. See Materials and methods section for more details. Mutations are all known pathogenic mutation described using HGVS nomenclature. (PDF) [file pone.0064268.s011.pdf]

Table S7. *BRCA1* classification results of lumB *BRCA1* (*n* =9) and lumB sporadic (*n* = 48) tumors obtained using leave-one-out cross-validation. See Materials and methods section for more details. Mutations are all known pathogenic mutation described using HGVS nomenclature.

SampleID	Group	Age	Prediction	WHO	Grade	Mutation	Functional effects
A086	*BRCA1*	28	Sporadic	IDC	1	*BRCA1* c.2475delC, Exon11, p.(Asp825Glufs*21)	FS
A016	*BRCA1*	39	*BRCA1*	IDC	1	*BRCA1* c.2476delA, Exon11, p.(Thr826Glnfs*20)	FS
A120	*BRCA1*	46	Sporadic	NA	NA	*BRCA1* c.2475delC, Exon11, p.(Asp825Glufs*21)	FS
A129	*BRCA1*	52	Sporadic	IDC	1	*BRCA1* c.115T>G, Exon3, p.(Cys39Gly)	MS
A133	*BRCA1*	57	*BRCA1*	IDC	3	*BRCA1* c.5266dupC, Exon20, p.(Gln1756Profs*74)	FS
A007	*BRCA1*	57	Sporadic	IDC	2	*BRCA1* c.5503C>T, Exon24, p.(Arg1835*)	NS
A125	*BRCA1*	62	*BRCA1*	IDC	3	*BRCA1* c.2475delC, Exon11, p.(Asp825Glufs*21)	FS
A170	*BRCA1*	72	Sporadic	IDC	3	*BRCA1* c.4987-?_5193+?del, Exon17-19, p.(Met1663_Glu1731del)	Exon del (IF)
A174	*BRCA1*	74	*BRCA1*	IDC	3	*BRCA1* c.4987-?_5193+?del, Exon17-19, p.(Met1663_Glu1731del)	Exon del (IF)
K058	Sporadic	36	Sporadic	IDC	3	-	
K046	Sporadic	43	Sporadic	IDC	3	-	
K083	Sporadic	43	Sporadic	IDC	3	-	
K079	Sporadic	43	Sporadic	IDC	3	-	
K011	Sporadic	44	Sporadic	IDC	3	-	
K123	Sporadic	44	Sporadic	IDC	2	-	
K088	Sporadic	47	Sporadic	IDC	2	-	
K074	Sporadic	48	Sporadic	ILC	2	-	
K021	Sporadic	49	Sporadic	IDC	2	-	
K056	Sporadic	50	Sporadic	IDC	1	-	
K113	Sporadic	50	Sporadic	IDC	1	-	
K114	Sporadic	50	*BRCA1*	IDC	2	-	
K057	Sporadic	51	Sporadic	IDC	2	-	
K064	Sporadic	51	Sporadic	IDC	2	-	
K089	Sporadic	53	Sporadic	IDC	2	-	
K055	Sporadic	54	*BRCA1*	Medullary	NA	-	
K052	Sporadic	58	*BRCA1*	IDC	3	-	
K148	Sporadic	60	Sporadic	IDC	3	-	
K082	Sporadic	60	Sporadic	IDC	2	-	
K155	Sporadic	61	Sporadic	IDC	2	-	
K043	Sporadic	63	Sporadic	IDC	2	-	
K031	Sporadic	64	Sporadic	IDC	2	-	
K086	Sporadic	64	Sporadic	IDC	2	-	
K108	Sporadic	64	Sporadic	ILC	NA	-	
K147	Sporadic	65	Sporadic	IDC	2	-	
K179	Sporadic	66	Sporadic	IDC	2	-	
K013	Sporadic	69	Sporadic	Other	NA	-	
K175	Sporadic	71	*BRCA1*	IDC	3	-	
K120	Sporadic	72	*BRCA1*	IDC	2	-	
K151	Sporadic	72	Sporadic	IDC	2	-	
K142	Sporadic	74	Sporadic	NA	NA	-	
K091	Sporadic	74	*BRCA1*	IDC	3	-	
K003	Sporadic	75	Sporadic	IDC	2	-	
K150	Sporadic	75	Sporadic	IDC	2	-	
K062	Sporadic	75	Sporadic	IDC	2	-	
K133	Sporadic	76	*BRCA1*	Other	NA	-	
K027	Sporadic	77	Sporadic	IDC	2	-	
K019	Sporadic	78	Sporadic	IDC	2	-	
K128	Sporadic	81	*BRCA1*	IDC	2	-	
K054	Sporadic	82	Sporadic	IDC	3	-	
K171	Sporadic	83	Sporadic	IDC	1	-	
K165	Sporadic	85	Sporadic	IDC	1	-	
K038	Sporadic	85	*BRCA1*	IDC	2	-	
K101	Sporadic	85	Sporadic	ILC	NA	-	
K080	Sporadic	86	Sporadic	Tubular	NA	-	
K066	Sporadic	87	*BRCA1*	Mucinous	NA	-	
K154	Sporadic	88	Sporadic	IDC	3	-	
K045	Sporadic	89	Sporadic	IDC	2	-	

Abbreviations: MS, missense mutation; FS, frameshift mutation; NS, nonsense mutation; IF, in-frame mutation
