# Supplementary material for: Classifications within Molecular Subtypes Enables Identification of BRCA1/BRCA2 Mutation Carriers by RNA Tumor Profiling
Source: PLoS One. 2013 May 21;8(5):e64268. doi: 10.1371/journal.pone.0064268 (PMC3660328; doi:10.1371/journal.pone.0064268)
Supplement: Table S8 — BRCA2 classification results of lumB BRCA2 ( n = 16) and lumB sporadic ( n = 48) tumors obtained using leave-one-out cross-validation. See Materials and methods section for more details. Mutations are all known pathogenic mutation described using HGVS nomenclature. (PDF) [file pone.0064268.s012.pdf]

Table S8. *BRCA2* classification results of lumB *BRCA2* (*n* =16) and lumB sporadic (*n* = 48) tumors obtained using leave-one-out cross-validation. See Materials and methods section for more details. Mutations are all known pathogenic mutation described using HGVS nomenclature.

SampleID	Group	Age	Prediction	WHO	Grade	Mutation	Functional effects
A013	*BRCA2*	28	Sporadic	IDC	3	*BRCA2* c.2830A>T, Exon11, p.(Lys944*)	NS
A109	*BRCA2*	31	*BRCA2*	IDC	2	*BRCA2* c.1310_1313delAAGA, Exon10, p.(Lys437Ilefs*22)	FS
A010	*BRCA2*	34	*BRCA2*	IDC	2	*BRCA2* c.2808_2811delACAA, Exon11, p.(Ala938Profs*21)	FS
A150	*BRCA2*	36	*BRCA2*	IDC	2	*BRCA2* c.6486_6489delACAA, Exon11, p.(Lys2162Asnfs*5)	FS
A095	*BRCA2*	37	*BRCA2*	IDC	3	*BRCA2* c.6490_6492delinsGACT, Exon11, p.(Gln2164Aspfs*12)	FS
A019	*BRCA2*	38	*BRCA2*	IDC	3	*BRCA2* c.6373delA, Exon11, p.(Thr2125Profs*12)	FS
A004	*BRCA2*	41	*BRCA2*	IDC	2	*BRCA2* c.2830A>T, Exon11, p.(Lys944*)	NS
A102	*BRCA2*	42	*BRCA2*	IDC	2	*BRCA2* c.7617+1G>A, Splice mut, Exon15 skipping	Exon skipping
A111	*BRCA2*	42	Sporadic	IDC	1	*BRCA2* c.1310_1313delAAGA, Exon10, p.(Lys437Ilefs*22)	FS
A117	*BRCA2*	45	*BRCA2*	IDC	2	*BRCA2* c.6601delT, Exon11, p.(Ser2201Leufs*5)	FS
A108	*BRCA2*	45	*BRCA2*	IDC	2	*BRCA2* c.6486_6489delACAA, Exon11, p.(Lys2162Asnfs*5)	FS
A003	*BRCA2*	48	*BRCA2*	IDC	2	*BRCA2* c.2830A>T, Exon11, p.(Lys944*)	NS
A106	*BRCA2*	60	*BRCA2*	IDC	3	*BRCA2* c.9015delA, Exon23, p.(Arg3005Serfs*23)	FS
A173	*BRCA2*	63	*BRCA2*	NA	NA	*BRCA2* c.8575delC, Exon20, p.(Gln2859Lysfs*4)	FS
A163	*BRCA2*	66	*BRCA2*	IDC	3	*BRCA2* c.3530_3533delACAG, Exon11, p.(Asp1177Alafs*19)	FS
A115	*BRCA2*	72	*BRCA2*	IDC	3	*BRCA2* c.2808_2811delACAA, Exon11, p.(Ala938Profs*21)	FS
K058	Sporadic	36	Sporadic	IDC	3	-	
K046	Sporadic	43	Sporadic	IDC	3	-	
K083	Sporadic	43	*BRCA2*	IDC	3	-	
K079	Sporadic	43	Sporadic	IDC	3	-	
K011	Sporadic	44	*BRCA2*	IDC	3	-	
K123	Sporadic	44	Sporadic	IDC	2	-	
K088	Sporadic	47	Sporadic	IDC	2	-	
K074	Sporadic	48	Sporadic	ILC	2	-	
K021	Sporadic	49	Sporadic	IDC	2	-	
K056	Sporadic	50	Sporadic	IDC	1	-	
K113	Sporadic	50	Sporadic	IDC	1	-	
K114	Sporadic	50	Sporadic	IDC	2	-	
K057	Sporadic	51	Sporadic	IDC	2	-	
K064	Sporadic	51	Sporadic	IDC	2	-	
K089	Sporadic	53	Sporadic	IDC	2	-	
K055	Sporadic	54	Sporadic	Medullary	NA	-	
K052	Sporadic	58	Sporadic	IDC	3	-	
K148	Sporadic	60	Sporadic	IDC	3	-	
K082	Sporadic	60	Sporadic	IDC	2	-	
K155	Sporadic	61	Sporadic	IDC	2	-	
K043	Sporadic	63	Sporadic	IDC	2	-	
K031	Sporadic	64	Sporadic	IDC	2	-	
K086	Sporadic	64	Sporadic	IDC	2	-	
K108	Sporadic	64	Sporadic	ILC	NA	-	
K147	Sporadic	65	Sporadic	IDC	2	-	
K179	Sporadic	66	Sporadic	IDC	2	-	
K013	Sporadic	69	Sporadic	Other	NA	-	
K175	Sporadic	71	Sporadic	IDC	3	-	
K120	Sporadic	72	Sporadic	IDC	2	-	
K151	Sporadic	72	Sporadic	IDC	2	-	
K142	Sporadic	74	Sporadic	NA	NA	-	
K091	Sporadic	74	*BRCA2*	IDC	3	-	
K003	Sporadic	75	Sporadic	IDC	2	-	
K150	Sporadic	75	Sporadic	IDC	2	-	
K062	Sporadic	75	*BRCA2*	IDC	2	-	
K133	Sporadic	76	Sporadic	Other	NA	-	
K027	Sporadic	77	Sporadic	IDC	2	-	
K019	Sporadic	78	Sporadic	IDC	2	-	
K128	Sporadic	81	*BRCA2*	IDC	2	-	
K054	Sporadic	82	Sporadic	IDC	3	-	
K171	Sporadic	83	Sporadic	IDC	1	-	
K165	Sporadic	85	Sporadic	IDC	1	-	
K038	Sporadic	85	Sporadic	IDC	2	-	
K101	Sporadic	85	Sporadic	ILC	NA	-	
K080	Sporadic	86	Sporadic	Tubular	NA	-	
K066	Sporadic	87	Sporadic	Mucinous	NA	-	
K154	Sporadic	88	Sporadic	IDC	3	-	
K045	Sporadic	89	Sporadic	IDC	2	-	

Abbreviations: FS, frameshift mutation; NS, nonsense mutation
