# Supplementary material for: Classifications within Molecular Subtypes Enables Identification of BRCA1/BRCA2 Mutation Carriers by RNA Tumor Profiling
Source: PLoS One. 2013 May 21;8(5):e64268. doi: 10.1371/journal.pone.0064268 (PMC3660328; doi:10.1371/journal.pone.0064268)
Supplement: Table S9 — The basal BRCA1 signature. 76 out of 110 genes were contained on the Rosetta chip used in the NKI study and 69 genes were present in the Jönsson dataset (indicated by ×). (PDF) [file pone.0064268.s013.pdf]

Table S9. The basal *BRCA1* signature. 76 out of 110 genes were contained on the Rosetta chip used in the NKI study and 69 genes were present in the Jönsson dataset (indicated by ×)

	NKI	Jönsson		NKI	Jönsson
AZGP1P1		x	OR2T2		
AZGP1	x		C6orf126		
NKX3-1	x		C11orf41	x	
FRMD4A	x	x	STK31		x
SYNM	x		IGSF5		
LOC100507507	x		FLJ22763		
PET112	x		NKD2		x
ZKSCAN4	x	x	CHRM1	x	x
CHD7	x		DHRS7B	x	x
NLK	x	x	DNMT3B	x	x
TUSC5		x	ALS2CL		x
PRKCA	x	x	FADS3	x	
TNFSF11	x	x	EIF2B5	x	x
KIAA0100	x	x	SWT1	x	
TMEM199	x	x	LARP6	x	x
EIF1AY	x	x	TDRD5		x
SIX2	x	x	ATP6AP2	x	x
ADAMTS20		x	C20orf94		x
EIF1AX			MAGEA8	x	x
NTRK2	x	x	METTL7A	x	x
JARID2	x	x	EPX	x	x
SFRP1	x	x	AQP7	x	x
LOC389023	x		NCRNA00162		
PCK1	x		SERPINA3	x	x
MAOB	x		SLC25A18	x	x
ERAL1	x	x	XLOC_014068		
SLC12A1	x	x	EIF5A2	x	x
CD36	x	x	EFHD1		x
MCTP2	x	x	DBI	x	x
B9D1	x	x	NCRNA00163		
MAOA	x	x	LGALS12	x	x
FRMD5	x	x	RETSAT	x	x
CSRP1	x	x	XLOC_004323		
DYNLT3	x	x	NEK3	x	x
PPP1R1A	x	x	KRTAP26-1		
LOC149773			TMEM130		
OGFRL1	x	x	C7orf46	x	x
COMMD3	x	x	CEP250	x	x
TRAF4	x	x	ITSN1	x	
HIGD1C			IFFO2	x	x
PYY2	x		EGLN1	x	x
TMEM26			LOC644366		
C21orf59	x		CASK	x	x
LOC442434			SPZ1		
ASB7	x	x	POPDC3		x
MAPK7	x	x	LOC100509860		
MYB	x	x	RPL23A	x	
DPP10	x		ST5	x	
FAM20C			MBOAT2	x	x
DMD	x	x	PDRG1	x	x
KREMEN1	x	x	MED6	x	x
MRPS36	x	x	HMGB3P22		
LAMC2	x	x	ZKSCAN3	x	x
TUBAL3			DENND2A		x
LOC100134285			TMEM200C		
