# Supplementary material for: Classifications within Molecular Subtypes Enables Identification of BRCA1/BRCA2 Mutation Carriers by RNA Tumor Profiling
Source: PLoS One. 2013 May 21;8(5):e64268. doi: 10.1371/journal.pone.0064268 (PMC3660328; doi:10.1371/journal.pone.0064268)
Supplement: Table S10 — The lumB BRCA2 signature. 77 out of the 100 genes were present in the Jönsson dataset (indicated by ×). (PDF) [file pone.0064268.s014.pdf]

Table S10. The lumB BRCA2 signature. 77 out of the 100 genes were present in the Jönsson dataset (indicated by ×)

	Jönsson		Jönsson
CCNA2	x	NKTR	x
SCYL2	x	PSMD3	x
DR1	x	EXOSC2	x
MRPS23	x	C1orf204	
PNPT1	x	DSC2	x
COQ2	x	TMPO	x
EIF4A3	x	RAB21	x
CDCA5	x	PATL1	x
SHCBP1	x	ZFAND2A	x
FEN1	x	ZBTB26	
MAD2L1	x	PPM1G	x
ANLN	x	EFTUD2	x
TIMM8A	x	XLOC_006922	
ASCC1	x	RABGEF1	x
OGT	x	TTC4	
CFL1	x	FRAS1	x
C12orf73		G3BP2	x
ZWINT	x	COG8	x
MRPL49	x	STRN	x
PSMD12	x	POP1	
EEF2K	x	MED24	x
POLR2G	x	IL20	x
UBE2NL		PILRB	x
COX6A1	x	CRADD	x
POLR2D		ESPNL	
DPH3	x	CBX3	
FBXW2	x	USP30	x
RBM18	x	CAPN1	x
MRPS7	x	LOC283911	
FEM1C	x	TBC1D7	x
RBM5	x	LOC441268	
TIMM10	x	ANAPC10	
LOC100131564		PSMA6	x
GATC	x	ATXN3	
GTPBP4	x	SCAF8	
C6orf97	x	IL24	x
FAM83D	x	TMEM181	x
RAN	x	ALDH6A1	x
RBMX2	x	CENPE	x
TTC3		GLT8D1	x
CNTF	x	TMF1	x
TEX19	x	FTSJ2	x
ZFP91	x	MRS2	x
ABHD14A	x	SDHAF2	x
METTL6		PPOX	x
BUB1	x	GTF2I	
INO80E	x	ANKRD30BL	
PION		KIAA0391	x
PDCL3		RAB11FIP2	x
PSMD14	x	FAT3	
