# Supplementary material for: Classifications within Molecular Subtypes Enables Identification of BRCA1/BRCA2 Mutation Carriers by RNA Tumor Profiling
Source: PLoS One. 2013 May 21;8(5):e64268. doi: 10.1371/journal.pone.0064268 (PMC3660328; doi:10.1371/journal.pone.0064268)
Supplement: Table S11 — Distribution of predicted molecular subtypes within the NKI dataset. (PDF) [file pone.0064268.s015.pdf]

Table S11. Distribution of predicted molecular subtypes within the NKI dataset

	**Basal-like**	**HER2-enriched**	**Luminal A**	**Luminal B**	**Normal-like**	**Total**
BRCA1	16	1	0	0	1	**18**
BRCA2	0	0	2	0	0	**2**
**Sporadic**	18	12	27	38	2	**97**
