# Supplementary material for: Classifications within Molecular Subtypes Enables Identification of BRCA1/BRCA2 Mutation Carriers by RNA Tumor Profiling
Source: PLoS One. 2013 May 21;8(5):e64268. doi: 10.1371/journal.pone.0064268 (PMC3660328; doi:10.1371/journal.pone.0064268)
Supplement: Table S12 — Distribution of predicted molecular subtypes within the Jönsson dataset. (PDF) [file pone.0064268.s016.pdf]

Table S12. Distribution of predicted molecular subtypes within the Jönsson dataset

	**Basal-like**	**HER2-enriched**	**Luminal A**	**Luminal B**	**Normal-like**	**Total**
BRCA1	13	0	1	1	2	**17**
BRCA2	4	1	5	21	0	**31**
**Non-*BRCA1/2***	19	19	40	38	8	**124**
**Sporadic**	34	31	26	68	13	**172**
