# Supplementary material for: Classifications within Molecular Subtypes Enables Identification of BRCA1/BRCA2 Mutation Carriers by RNA Tumor Profiling
Source: PLoS One. 2013 May 21;8(5):e64268. doi: 10.1371/journal.pone.0064268 (PMC3660328; doi:10.1371/journal.pone.0064268)
Supplement: Table S13 — BRCA1/2 classification results using the standard clinical variables ER, PR, HER2, TNBC (ER−/PR−/HER2−) and age of onset of disease. (PDF) [file pone.0064268.s017.pdf]

Table S13. BRCA1/2 classification results using the standard clinical variables ER, PR, HER2, TNBC (ER-/PR-/HER2-) and age of onset of disease.

	No. of samples	Sensitivity (TP)	Specificity (TN)	Accuracy[a]	*p*-value[b]
***BRCA1* vs. Sporadic**					
ER	33 vs. 128	0.58 (19)	0.84 (107)	0.71	5.1×10^{-6}
PR	33 vs. 128	0.79 (26)	0.62 (79)	0.70	3.2×10^{-5}
HER2	33 vs. 128	0.91 (30)	0.16 (21)	0.54	4.1×10^{-1}
Age of onset (>50 years)	33 vs. 128	0.64 (21)	0.80 (103)	0.72	2.1×10^{-6}
***BRCA2* vs. Sporadic**					
ER	22 vs. 128	0.09 (2)	0.84 (107)	0.46	5.3×10^{-1}
PR	22 vs. 128	0.27 (6)	0.62 (79)	0.44	4.7×10^{-1}
HER2	22 vs. 128	0.05 (1)	0.84 (107)	0.44	2.0×10^{-1}
Age of onset (>50 years)	22 vs. 128	0.77 (21)	0.80 (103)	0.79	2.3×10^{-7}
***Basal-like BRCA1* vs. Basal-like Sporadic**					
Age of onset (>50 years)	20 vs. 10	0.70 (14)	1.00 (10)	0.85	3.0×10^{-4}
***LumB BRCA1* vs. LumB Sporadic**					
Age of onset (>50 years)	9 vs. 48	0.33 (3)	0.75 (36)	0.54	6.9×10^{-1}
***LumB BRCA2* vs. LumB Sporadic**					
Age of onset (>50 years)	16 vs. 48	0.75 (12)	0.75 (36)	0.75	6.8×10^{-4}

[a] Mean balanced accuracy

[b] Fisher’s exact test

Abbreviations: TP, true positive; TN, true negative
